# Olfactory spatial memory: a systematic review and meta-analysis

**DOI:** 10.1038/s41598-025-25503-5

**Published:** 2025-11-04

**Authors:** Malina Szychowska, Jonas K. Olofsson, Nira Cedres

**Affiliations:** 1 Department of Psychology, Stockholm University, Stockholm, Sweden; 2 Facultad de Ciencias de la Salud, Universidad Fernando Pessoa Canarias, Las Palmas de Gran Canaria, Spain

**Keywords:** Olfactory spatial memory, Odor-place association, Odor-cued navigation, Olfactory landmarks, Cognitive maps, Olfactory system, Spatial memory, Human behaviour

## Abstract

The human sense of smell is extraordinarily sensitive, and some argue that a main purpose of olfaction is to localize food sources. Yet, little is known about human spatial memory for odors and the existing literature has never been systematically summarized. In this systematic review and meta-analysis, we evaluated the evidence regarding human abilities to memorize arrangements of objects or routes based on olfactory cues, and compared results to those of other senses. We followed the PRISMA guidelines, searching three databases (PubMed, WoS, Scopus). Using manual and automated (ASReview) screening methods, we identified 24 articles that complied with our inclusion criteria (e.g., olfactory spatial memory task, healthy human adults). Four key results emerged. First, odors can be used as landmarks in the formation of neuro-cognitive maps in both sensory and cognitive brain areas, providing a foundation for spatial memory and navigation abilities. Second, the human ability to memorize locations of odors, especially cues signaling high-calorie food, is indicative of its presumed evolutionary role in foraging. Third, odor-recognition and odor-context-place association might stem from overlapping memory processes. Fourth, olfactory targeted training enhances olfactory spatial memory and shows transfer to other modalities and cognitive domains. Seven articles that compared performances in olfactory versus visual spatial memory were eligible for meta-analysis. The analysis was carried out using the standardized mean difference (i.e., Glass’s estimator) as the outcome measure. A random-effects model was fitted to the data. Results showed that olfactory spatial memory is less effective than visual memory ($\:\widehat{\mu\:}$ = −0.48, *95% CI* [ − 0.77, − 0.19]). Notably, two studies exhibiting the largest differences between sensory modalities were either evaluated as of moderate quality or included older participants. Furthermore, all included studies varied in the methods and materials used. Thus, we suggest that addressing methodological variations across studies and investigating the neural mechanisms will be pivotal for further advancing this research field.

**Supplementary Information:**

The online version contains supplementary material available at 10.1038/s41598-025-25503-5.

## Introduction

### Rationale

 Human episodic memories are intrinsically defined in space and time^1^. The spatial memory and navigation system of the mammalian brain is now a relatively well-understood neurocognitive domain, and it was the subject of the 2014 Nobel Prize in Physiology or Medicine. Spatial memory can be defined as a “brain function responsible for recognizing, codifying, storing and recovering spatial information about the arrangement of objects or specific routes”^2^. A common assumption in psychological science is that cognitive abilities are domain-general. Often, implicit assumptions are made when generalizing cognitive findings from the sensory system in which it was researched (e.g., making conclusions about “working memory” from studying only visual working memory). However, there is in fact scarce evidence on whether human spatial memory interacts similarly with different sensory modalities, and whether findings related to spatial memory can be generalized across all senses in humans. In research on human participants, the literature is dominated by vision^3^, even though research on rodents often involves the sense of smell. Although one should always be mindful of the profound differences between species when discussing any physiological or sensory/cognitive function, the olfactory system in rodents shares many similarities with the human olfactory system^4^and often provides models for human cognitive abilities e.g^4–7^.,.

Recent evidence indicates that olfaction is underrepresented in research on human spatial navigation and memory^8^, but spatial processes might be uniquely influenced by olfaction. Human olfaction stands out by having a uniquely direct (unmediated by a thalamic relay) and strong neuroanatomical connection to higher processing centers^9^. Olfaction operates through a cortical network extending from the olfactory bulb to the primary olfactory regions in the medial temporal lobe^9^, which are in turn strongly connected to the core spatial memory and navigation system, including the hippocampus (HC) and entorhinal cortex (EC)^9–11^. Moreover, there is an apparent neuroanatomical integration of perceptual and cognitive functions in olfaction, where cognitive areas aid perceptual tasks, and perceptual areas engage in cognitive tasks. Indeed, the HC and EC contribute to olfactory tasks such as odor identification (i.e., matching familiar odors to corresponding labels^12,13^. Previous studies showed that human visual spatial abilities correlate with olfactory identification performance, and volume of the HC predicts both olfactory functions and spatial memory functions^14–16^. Likewise, the primary olfactory areas engage in smell-related cognitive tasks, such as mental navigation in conceptual olfactory space^17^ or odor-cued spatial navigation tasks^18^. Furthermore, cognitive maps in the EC-HC network, which may provide a foundation for spatial memory and navigation ability, were recently recorded in humans using olfactory landmarks in navigation tasks^18^, and amnesic patients with HC damage are unable to associate specific smells with their correct locations in an odor-place association task^19^. Finally, some argue that the ability to remember locations of smells might be evolutionarily beneficial because it helps locating and navigating to food sources^20,21^. It has even been suggested that olfaction might have evolved primarily to aid spatial navigation, so that spatial orientation is its primary function^22,23^ and that olfaction should indeed be regarded as a spatial sense^24^. Notably, there is a growing interest in studying olfactory spatial memory, shown by a steady increase in the number of publications related to that topic over the last 20 years (Supplementary Fig. 1). Therefore, it is of interest to summarize the current state of knowledge about human olfactory spatial memory, to compare whether human olfactory spatial memory is as good as other sensory modalities, or maybe even better.

We systematically reviewed the evidence regarding human abilities to memorize the arrangement of objects or routes based on olfactory cues, and we compared olfactory spatial memory performance with that of other senses qualitatively and in a meta-analysis. Notably, although the individual studies might focus on many different aspects of human olfaction or memory, we emphasize aspects that are of particular relevance to our theoretical viewpoint, as outlined above.

### Objectives

To systematically evaluate human ability to memorize locations of smells and use them in spatial navigation tasks, and to test whether olfactory spatial memory abilities differ from those of other senses.

## Methods

The present study was conducted in line with the PRISMA statement that outlines recommendations and guidelines for systematic reviews and meta-analyses^25^. The study was not preregistered. All data for this project, along with supplementary material, relevant code, and codebook with metainfo, have been made publicly available and can be accessed on our OSF repository (https://osf.io/5y9vj/)^26^.

### Information sources and search strategy

Three databases (PubMed, Web of Science, Scopus) were searched on the 10th of September 2024 to identify the relevant studies. Search terms were selected in line with the PICO (Population, Intervention, Comparison, Outcome) framework. Search terms were partially identified by utilizing ChatGPT (GPT-3) to provide synonyms of phrases that could be used in relation to odor, olfaction, and spatial memory. Example search terms are: Adult population, healthy adults, odor, olfaction, olfactory sensation, scent, cognitive mapping, location memory, object-place association (for all search terms see Supplement Table 1 in “Supplement_1_tables_and_figures.pdf”). Additionally, a filter limiting the language of studies to English was applied. Search strategies for the three databases are available in Supplement Table 2.

### Eligibility criteria

Spatial memory may be treated either as implicit or explicit in nature. Some cases in real life where spatial memory guides behavior does not require conscious recall, but often spatial memories are evoked by explicit questions that require conscious recall (e.g., “Where did I leave my phone?”). Notably, the concept of spatial memory in this review does not include studies that tested memories of fully implicit nature, where participants were neither asked to memorize location of smells, nor tested on their memory specifically e.g^27,28^.,. Instead, we included studies that allow for a direct investigation of olfactory spatial memory through conscious memory recall. More inclusion and exclusion criteria are presented in Table 1.

**Table 1:** Inclusion and exclusion criteria used to select the relevant studies, divided into four categories: study design, Aim, Population, Outcome.

	Inclusion criteria	Exclusion criteria
Study design	• Spatial memory task • Empirical study	• Review • Case report • Editorial • Opinion paper
Aim	• Olfactory spatial memory evaluation • Comparison between olfactory and other sensory spatial memory	
Population	• Healthy human adults • Control sample from clinical studies	• Clinical populations • Animals • Children
Outcome	• Olfactory spatial memory • Olfactory object-place association • Olfactory navigation memory	• Studies investigating non-olfactory memory • Studies investigating non-spatial aspects of memory

### Study selection

**Fig. 1:**
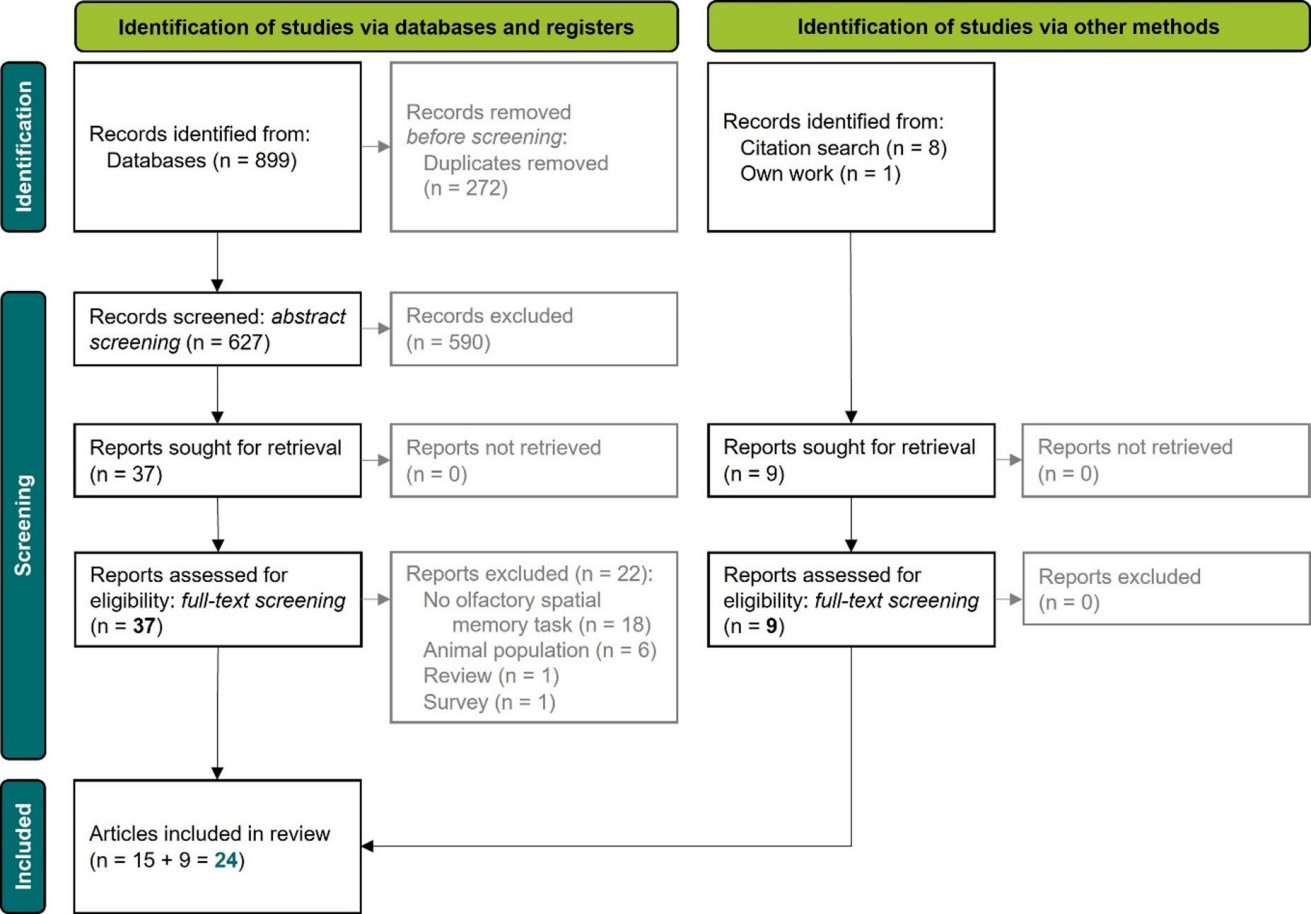
PRISMA 2020 flow diagram for new systematic reviews which included searches of databases, registers and other sources^25^.

Our search strategy resulted in identification of 899 articles, out of which 272 were duplicates. After abstract (*n* = 627 articles) and full-text (*n* = 37 articles) screening, 15 articles were selected for the systematic review. During full-text screening of the relevant 15 articles and manuscript preparation, additional eight relevant articles were discovered by reference tracking (for identification of articles discovered with search and reference tracking, see Supplement Table 3). These articles were screened and added to the systematic review. Also, one additional peer-reviewed empirical article, which was published during preparation of this manuscript, was added to the systematic review. Together, 24 articles are included in the present review (Fig. 1). List of articles excluded during full-text screening and reason for exclusions is available in Supplement Table 4.

### Selection process

All studies were screened at two stages (title and abstract, and full text screening) by one reviewer (MS). Uncertainties were consulted with the other reviewer (NC) leading to a consensus decision. During the title and abstract screening process, articles were assessed manually and, in parallel, utilizing artificial intelligence with the ASReview application, a free-source tool, created to make the article screening process faster and more efficient^29,30^. For more details and analytic insights from the automated screening process, see “Supplement_2_ASReview_details.pdf” on OSF^26^.

### Data collection process

We found 15 studies that compared performance in the spatial memory task between sensory modalities, including olfaction (either between- or within-group design). Thirteen studies compared performance in olfaction with that in vision, four with hearing, one with touch, one with a combination of olfaction and semantics (i.e., participants first heard the list of odor names, then were presented with a sequence of odors at specific locations, and during recall were tasked with replicating that sequence while identifying the odors using previously heard list), two with a combination of olfaction and vision, one with partial sensory deprivation (no hearing, vision, or olfaction), and one with multimodal perception (vision, olfaction, and taste). One reviewer (MS) extracted data from all these studies. Besides the olfactory-visual comparison, only qualitative syntheses could be derived from the retrieved data in other modalities, because the studies were too few to perform meta-analyses. However, we retrieved enough data regarding the differences between olfaction and vision to perform a meta-analysis of how olfactory spatial memory may differ from visual spatial memory. Note that not all studies provided raw data or group means and standard deviations (*SD*) in text or table for the relevant variables, but some provided supplementary files with data or showed data on the plots. If no other source of data was available, the reviewer (MS) used a screen measuring ruler application (Page Ruler add-on to Google Chrome browser) and proportion calculations to estimate relevant values from the plots. For studies that reported standard error of the mean (*SEM*) or 95% confidence interval (*95% CI*), *SD* was calculated using appropriate formulas (see script “Supplement_metaanalysis.R”^26^).

### Data items

Data were extracted from the studies that compared spatial memory performance between olfaction and other conditions. Outcome variables of interest were: performance in a spatial memory task in the olfactory condition, and performance in a spatial memory task in the other sensory condition. Performance could be measured in multiple ways, for example number of correctly placed objects, proportion of correct object-place associations, distance error, etc. Any measure of performance was deemed eligible for inclusion. If a study reported results for clinical and control samples, only the control sample was taken into account. If a study reported results divided into groups of participants, and these groups were non-clinical samples (e.g., athletes and non-athletes, younger and older, etc.) combined results for the groups were estimated and included in the meta-analysis. Formulas for pooled means and SDs can be found in the “Supplement_1_tables_and_figures.pdf”.

### Study risk of bias assessment

Risk of bias in the included studies was assessed by one reviewer (MS) using a selection of questions from the “Checklist for analytical cross-sectional studies”^31^, in order to evaluate methodological quality of the studies. Uncertainties were consulted with the other reviewer (NC) leading to a consensus decision. For each study we calculated a score representing a sum of satisfied criteria (max 6 points).

### Statistical analysis

Data was prepared, processed, and visualized in R^32^ and RStudio^33^ utilizing mostly the tidyverse package (version 2.0.0)^34^. Meta analysis was performed using the package “metafor” (version 4.2-0.2)^35^. Scripts are available in the repository^26^. Because the included studies reported various types of performance measures, the analysis was carried out using the standardized mean difference (i.e., Glass’s estimator obtained with metafor::escalc()) as the outcome measure. A random-effects model was fitted to the data. The amount of heterogeneity (i.e., tau^2^, was estimated using the Hedges’ estimator. In addition to the estimate of tau^2^, we report the Q-test for heterogeneity and the l^2^ statistic. To examine whether studies may be outliers and/or influential in the context of the model, we evaluated the studentized residuals and Cook’s distances, and visually inspected the funnel plot.

## Results

### Study characteristics

#### Demographics summary

**Fig. 2:**
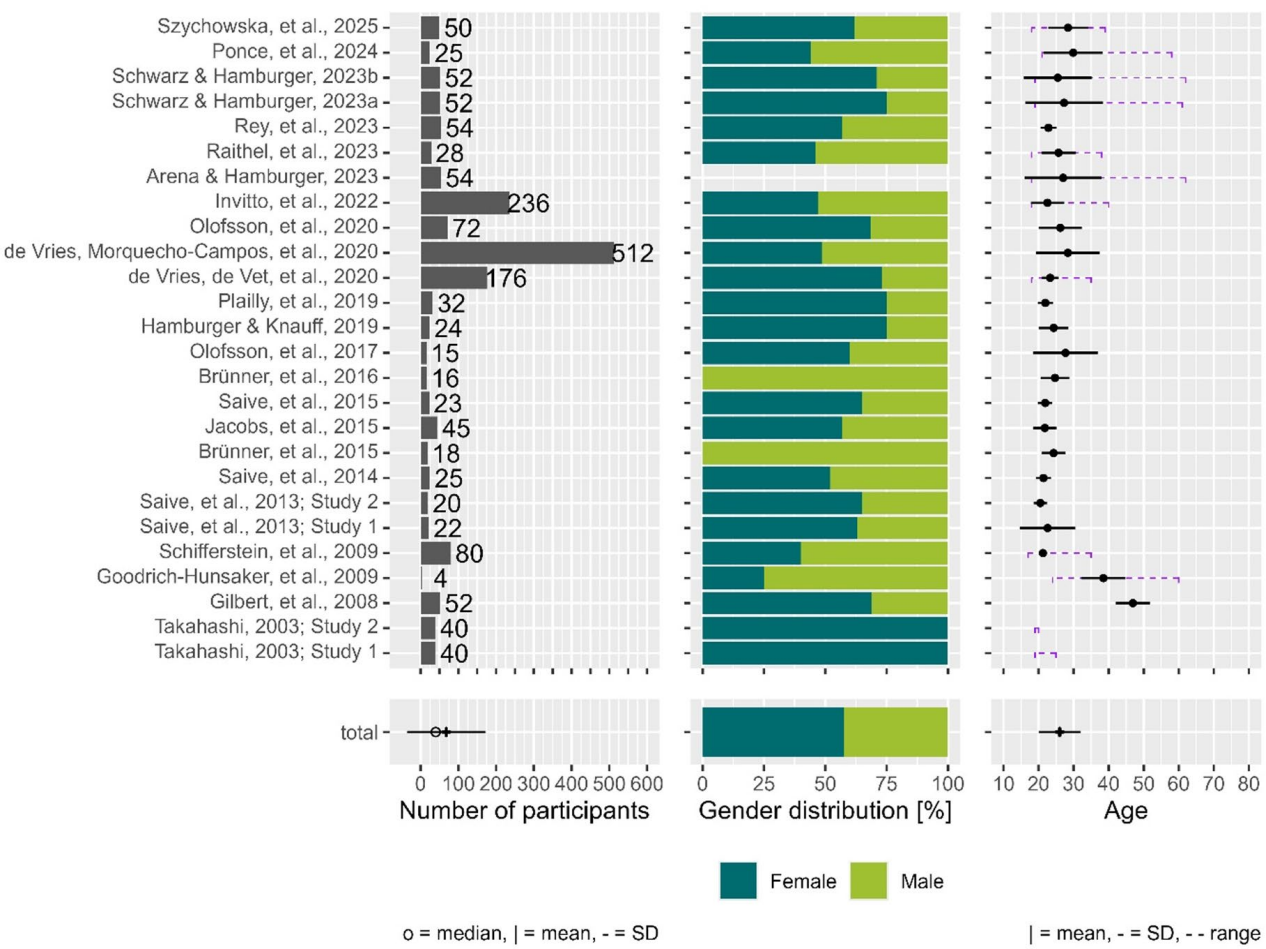
Summary of demographic information for individual studies (top panel) and across the studies (bottom panel). The left column shows the number of participants in individual studies in the top panel, and median (open circle), mean and SD across all studies in the bottom panel. The middle column shows color-coded gender distribution in individual studies (top), and averaged across all studies (bottom). The right column shows the mean, SD (solid line), and range (purple dashed line) of participants’ age in individual studies (top), and a mean and SD across all studies that provided mean age values (bottom). Note that two articles reported results from more than one study^36,37^, and in those instances, individual studies appear in separate rows in this figure.

Figure 2 depicts demographic characteristics of the total population in each study (across all experimental groups) and average across studies. For detailed demographic summary per experimental group within each study, see Supplementary Fig. 1. On average, studies tested 68 participants (*SD* = 104, *median* = 40), 57.5% of participants were female, and average age of participants was 26 years old (*SD* = 5.81). Most of the studies reported mean and SD or standard error of the mean (SEM) for the age, but two only reported participants’ age range^36^. For the studies that reported demographic information divided into different sample groups but did not provide a global summary, the pooled means and SDs for age shown on Fig. 2 were calculated according to the formulas presented in the supplementary material. Detailed description of demographic characteristics is available in the supplementary material.

#### Task summary

**Fig. 3:**
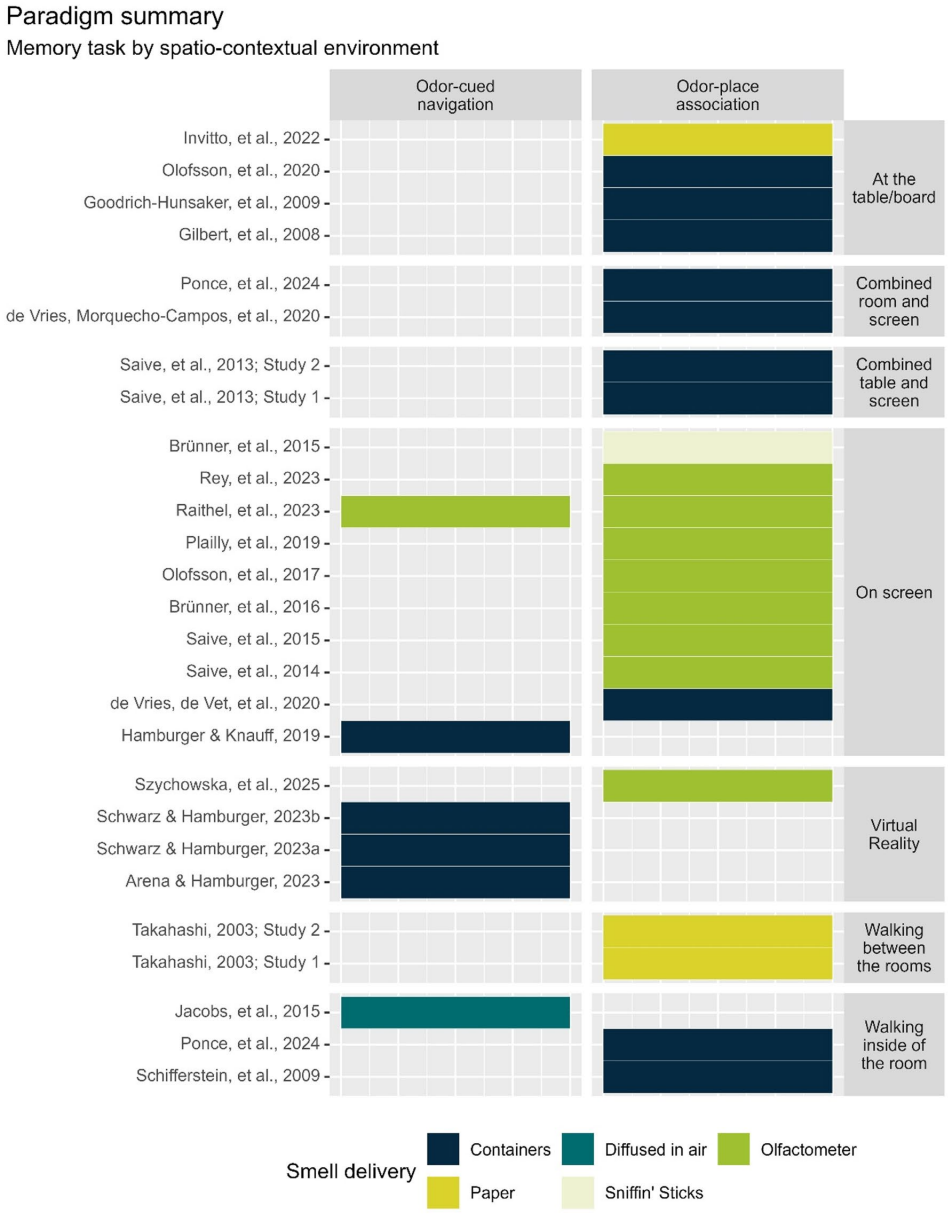
Summary of the methods used in the studies for three main variables: Memory task (odor-cued navigation or odor-place association) shown as separate columns, Spatio-contextual environment shown as separate blocks of rows, and Smell delivery marked with color coding. Note that two articles reported results from more than one study^36,37^, and the individual studies appear in separate rows in this figure. Furthermore, one study tested participants in two spatio-contextual settings and therefore appears twice in the figure^42^.

Figure 3 shows a summary of methods in the selected studies. In this review, we distinguish between odor-place association tasks, where participants were specifically asked to indicate correct odor-place association; and other tasks, which we decided to classify as odor-cued navigation tasks, where participants were asked to make a spatial decision based on an odor encountered on their way (e.g., turn left, move forward, stop, etc.). We adopted this distinction based on previous reviews of spatial memory that also distinguished between these two tasks, suggesting possibly different memory mechanisms for encoding object locations and routes due to additional sequential information present in route encoding^2,38^. Additionally, recalling routes might be based on associating a given stimulus with a spatial action^6^ rather than employing a specific spatial map or sequential information, further differentiating the two types of tasks. We acknowledge that our classification is limited, and some studies fit into these schemas better than others. More detailed division could be needed in the future to create more specific tasks categories, for example, those with continuous versus discrete smell sampling, or those using labyrinths versus “open field” navigation. For the purpose of this review, we propose to classify the found studies only into the two task types as a balance between grouping overly different studies together, and splitting them into too many task categories with only one or two studies in each.

Twenty-one studies (from 19 distinct scientific articles) employed an odor-place association task^18–21,36,37,39–51^ but the specific task designs and outcome measures varied, as shown on Fig. 3; Table 2 in section “Results of individual studies”. Note that on Fig. 3 there are 22 items marked as Odor-place association instead of 21, because in one study participants were tested in two settings, both while walking in the room and combined room and screen^42^. Furthermore, note that one study used a design that matched both task types^18^.

Six studies used different paradigms that we considered a type of an odor-cued navigation task^18,52–56^. Specifically, tasks were to: find a way in a virtual labyrinth (on screen) by making turning decisions based on olfactory (or visual, or olfacto-visual) landmarks^52–55^; find a location in the room where the perceived smell mixture matches previously encoded target, navigating based on the gradient of two odors^56^; and navigate to a specific odor location (odor-place association) using only olfactory landmarks (odor-cued navigation) in a virtual arena^18^.

#### Spatio-contextual environments summary

We identified seven different spatio-contextual environments that were used in the studies. In ten studies participants performed the memory task on a screen^18,20,44–50,55^. Notably, it was unclear whether Raithel et al.^18^ had presented the visual context on a computer screen or through the VR goggles, but because the study also included functional Magnetic Resonance Imaging (fMRI), we assumed the task was on the screen. In four studies, participants performed the task at a table or a board in front of them^19,39–41^; In two studies (described in one article) participants had to memorize odor locations on a board in front of them, and at the same time associate them with a visual context presented on the computer screen^37^; In three studies, participants were walking inside a room to perform the task^21,42,56^; In two studies (described in one article) participants were walking between two rooms^36^; In two studies, the location of objects were encoded inside a physical space, but recalled on a computer screen^42,43^. Finally, in four studies, participants performed the task in the Virtual Reality environment, wearing a VR headset^51–54^.

#### Odor presentation summary

We identified five different ways, in which odorants were presented to the participants, (Fig. 3). Thirteen studies (in 12 articles) used some type of odor container, such as glass jars or bottles^19,20,37,41–43,52–55^, tin-cans^40^, or custom designed SensaCubes^21^. Eight studies used an olfactometer^18,45–51^, three studies (described in two articles) used paper strips^36,39^, one study used Sniffin’ Sticks^44^, and in one study participants smelled odorants diffused in the air^56^. List of smells used in different studies is available in the supplementary material (see Supplement Table 6 and Supplement Figs. 4–7 in Supplement_1_tables_and_figures.pdf for odors from studies included in the meta-analysis, and “Supplement_SR_olfactory_spatial_memory.xlsx” for details on all studies).

### Risk of bias in studies

Risk of bias was assessed to evaluate methodological quality with established criteria^31^. Out of 24 studies, 21 were considered high quality (5–6 points), and three were considered medium quality (3–4 points). Specifically for the medium quality studies: two studies lacked information about the inclusion criteria, and detailed sample and design description^21,48^; and one (lowest scoring) study lacked information about the confounding factors and strategies to deal with those confounders, and outcome measures for different sensory modalities were obtained in a slightly different way^39^. Detailed assessment of risk of bias is available in Supplement Table 5.

### Results of individual studies

Results are grouped into tables, one reporting on olfactory spatial memory tasks in all the studies and providing detailed results for different conditions or sessions (Table 2), and two reporting only on the studies that compared olfactory spatial memory with other sensory conditions (Table 3 shows comparison between olfaction and vision, Table 4 shows comparison between olfaction and other modalities). Detailed qualitative report for individual studies can be found in the supplementary material (“Supplement_3_qualitative_report.pdf”).

Table 2 summarizes results in olfactory spatial memory tasks, including methods, outcomes, participants, and means and SD scores for all conditions and sessions. For studies that used a repeated measures design and tested participants over several sessions, and for studies that tested olfactory spatial memory under different conditions (e.g., food-related and non-food-related odors), results from distinct sessions/conditions are noted in individual rows. For articles that presented results from multiple studies/experiments, each study is shown as an individual observation.

**Table 2:** Summary of methods and results for olfactory Spatial memory.

Study authors (year)	Main task	Smell delivery	Spatial context	Explicit memorization instructions	Outcome measure	*N*	Additional conditions/sessions/comments	M	SD	Reported chance level
Arena & Hamburger (2023)^52^	Odor-cued navigation	Containers	Virtual reality	Unclear but most likely yes	Proportion of correct route decisions based on olfactory landmarks (larger = better)	17	Immediate recall	0.64	0.16	0.33
						16	Delayed recall (1 month)	0.55	0.15	0.33
						16	Combined	0.60	0.16	0.33
*Brünner et al. (2015)^44^	Odor-place association	Sniffin’ Sticks	On screen	Yes	Number of correct odor-place associations (larger = better)	18	Food (max 4) Repetition 1 Repetition 2 Repetition 3 Repetition 4 (delayed)	2.05 2.66 3.10 2.65	1.27 0.93 0.80 1.02	NA
							Non-food (max 4) Repetition 1 Repetition 2 Repetition 3 Repetition 4 (delayed)	1.44 1.99 2.39 2.27	1.15 1.36 1.27 1.27	NA
							Combined (max 8) Repetition 1 Repetition 2 Repetition 3 Repetition 4 (delayed)	3.49 4.66 5.51 4.83	2.08 1.78 1.82 1.82	NA
*Brünner et al. (2016)^45^	Odor-place association	Olfactometer	On screen	Yes	Number of correct odor-place associations (max 8, larger = better)	16	Maze 1	2.85	1.56	2.66
							Maze 2	3.46	1.92	2.66
							Combined (pooled)	3.16	1.75	2.66
de Vries, de Vet, et al. (2020)^20^	Odor-place association	Containers	On screen	No *(But explicit instruction to recall)*	Distance error from correct location [pixels] (smaller = better) *(Note: Means and SD calculated from the supplementary data files.)*	88	High-calorie content	118.27	69.13	NA
							Low-calorie content	152.72	85.58	NA
							Sweet	149.25	79.12	NA
							Savory	121.73	70.97	NA
							Combined	135.49	61.94	NA
de Vries, Morquecho-Campos, et al. (2020)^43^	Odor-place association	Containers	Combined room and screen	No *(But explicit instruction to recall)*	Proportion of correct relocations (larger = better)	254	High-calorie content	0.36	0.24	NA
							Low-calorie content	0.30	0.28	NA
							Combined	0.36	0.24	NA
*Gilbert et al. (2008)^41^	Odor-place association	Containers	At the table	Yes	Number of errors in the olfactory task (max 6; smaller = better)	13	Young adults	0.98	1.16	NA
						13	Older adults	3.00	2.16	NA
						26	Combined (estimated)	1.99	1.74	NA
*Goodrich-Hunsaker et al. (2009)^19^	Odor-place association	Containers	At the table/board	Yes	Number of correct odor-location associations (max 6; larger = better)	4	*(Note: the scale on the* Fig. 2A *in Goodrich-Hunsaker and colleagues (2009) contains an error*,* so 4.33 is just an approximate estimate)*	4.33	0.78	NA
					Number of “incorrect pair” errors (smaller = better)		-	1.25	0.83	NA
					Number of location errors (smaller = better)		-	0.75	0.21	NA
Hamburger & Knauff (2019)^55^	Odor-cued navigation	Containers	On screen	Yes	Proportion of correct route decision based on olfactory landmarks (larger = better)	24	-	0.64	0.19	0.33
Invitto et al. (2022)^39^	Odor-place association	Paper	At the table	Yes	Span in Corsi Test (larger = better)	153	Non-athletes	4.61	2.19	NA
						83	Athletes	3.37	2.10	NA
						236	Combined	4.17	2.16	NA
Jacobs et al. (2015)^56^	Odor-cued navigation	Diffused in air	Walking inside of the room	Yes	Distance error from correct location [cm] (smaller = better)	45	-	289	146	529.4
					Navigation time [s] (smaller = better)		-	79.9	62.8	NA
*Olofsson et al. (2017)^48^	Odor-place association	Olfactometer	On screen	Yes	Proportion of hits in the memory game (larger = better)	13	Before olfactory training	0.42	0.08	NA
							After olfactory training	0.53	0.09	NA
Olofsson et al. (2020)^40^	Odor-place association	Containers	At the table	Yes	N trials needed to complete the game (smaller = better)	41	Before olfactory training	38.1	10.8	NA
							After olfactory training	28.3	7.3	NA
						31	Before visual training	40.1	10.5	NA
							After visual training	38.5	9.6	NA
						72	Before training, combined	39	10.7	NA
							After training, combined	32.7	8.4	NA
Plailly et al. (2019)^47^	Odor-place association	Olfactometer	On screen	No *(But explicit instruction to recall)*	Proportion of correct associations in four episodic combinations (larger = better)	32	WWW = odor-context-place	0.17	0.19	0.019
							WWhere = odor-place	0.02	0.00	0.037
							WWhich = odor-context	0.22	0.01	0.148
							What = odor	0.36	0.03	0.296
Ponce et al. (2024)^42^	Odor-place association	Containers	In room	Yes	Number of correct object-place associations (max 8, larger = better)	25	In-room recall *(Note: Means and SD for in-room recall are provided by the authors after email exchange*,* in parenthesis are Medians and IQR values from the article.)*	7.44 (8)	1.04 (1)	NA
					Number of attempts to place objects (max 3 per stimulus, together max 24, larger = better)			2.64 (1)	3.68 (4)	NA
					Learning time [s] (smaller = better)			310 (300)	133 (300)	NA
					Replace time [s] (smaller = better)			402 (360)	122 (162)	NA
			On screen	Yes	Number of correct object-place associations (max 8, larger = better)	25	On-screen recall (only median and IQR available)	NA (6)	NA (3)	NA
*Raithel et al. (2023)^18^	Odor-cued navigation and odor-place association	Olfactometer	On screen	Yes	Number of completed trials (max ~ 90, larger = better)	28	Day 1	54.57	2.55	NA
							Day 2	70.85	2.55	NA
							Day 3	77.87	1.91	NA
							Day 4	79.15	1.28	NA
					Path tortuosity (smaller = better, min = 1)		Day 1	1.69	0.09	NA
							Day 2	1.33	0.07	NA
							Day 3	1.20	0.05	NA
							Day 4	1.19	0.03	NA
Rey et al. (2023)^46^	Odor-place association	Olfactometer	On screen	No *(But explicit instruction to recall)*	Probability to reach each episodic step [%] (larger = better)	54	Cue presentation to recognition	79.9	3.8	50
							Cue presentation to context (room) recall	-	-	NA
							Cue presentation to place (box) recall	19.2	2.4	5
							Cue recognition to context recall	51.5	3.0	33
							Cue recognition to place recall	26.0	2.9	11
							Room retrieval to place recall	52.2	4.3	33
*Saive et al. (2013)^37^ Study 1	Odor-place association	Containers	Combined table and screen	No *(But explicit instruction to recall)*	Number of correct associations, in four combinations (max 9, larger = better)	22	WWW = odor-context-place	3.56	2.0	NA
							WWhere = odor-place	0	0	NA
							WWhich = odor-context	0.83	1.22	NA
							What = odor	3.28	1.33	NA
*Saive et al. (2013)^37^ Study 2	Odor-place association	Containers	Combined table and screen	No *(But explicit instruction to recall)*	Proportion of correct associations, in four combinations, presented in five repetitions (larger = better)	20	WWW = odor-context-place Repetition 1 Repetition 2 Repetition 3 Repetition 4 Repetition 5	0.27 0.30 0.36 0.34 0.35	0.17 0.18 0.21 0.22 0.25	NA
							WWhere = odor-place Repetition 1 Repetition 2 Repetition 3 Repetition 4 Repetition 5	0.02 0.04 0.04 0.03 0.03	0.04 0.07 0.10 0.10 0.07	NA
							WWhich = odor-context Repetition 1 Repetition 2 Repetition 3 Repetition 4 Repetition 5	0.12 0.05 0.07 0.07 0.09	0.11 0.10 0.16 0.12 0.11	NA
							What = odor Repetition 1 Repetition 2 Repetition 3 Repetition 4 Repetition 5	0.41 0.44 0.42 0.37 0.39	0.14 0.17 0.22 0.20 0.26	NA
*Saive et al. (2014)^49^	Odor-place association	Olfactometer	On screen	No *(But explicit instruction to recall)*	Proportion of correct associations, in four combinations, for pleasant, neutral, and unpleasant odors (larger = better)	25	WWW = odor-context-place Pleasant Neutral Unpleasant Combined	0.32 0.17 0.35 0.26	0.31 0.21 0.32 0.15	0.18 0.18 0.18 0.018
							WWhere = odor-place Pleasant Neutral Unpleasant Combined	- - - 0.01	- - - 0.04	NA NA NA 0.02
							WWhich = odor-context Pleasant Neutral Unpleasant Combined	0.25 0.20 0.22 0.22	0.22 0.16 0.27 0.13	0.15 0.15 0.15 0.13
							What = odor Pleasant Neutral Unpleasant Combined	0.24 0.31 0.23 0.26	0.21 0.21 0.23 0.11	0.31 0.31 0.31 0.30
Saive et al. (2015)^50^	Odor-place association	Olfactometer	On screen	No *(But explicit instruction to recall)*	Number of correct associations, in four combinations (max 9, larger = better)	23	WWW = odor-context-place	3.09	1.31	0.019
							WWhere = odor-place	-	-	0.037
							WWhich = odor-context	1.35	0.88	0.148
							What = odor	3.09	1.53	0.296
Schifferstein et al. (2009)^21^	Odor-place association	Containers	Walking inside of the room	No *(But explicit instruction to recall)*	Proportion of correct relocations (larger = better)	20		0.22	0.16	0.10
Schwarz & Hamburger (2023a) *(Implicit versus…)*^54^	Odor-cued navigation	Containers	Virtual reality	Yes	Proportion of correct route decisions based on olfactory landmarks (larger = better) *(Note: Implicit here means after not recognizing the stimulus*,* explicit means after recognizing the stimulus)*	16	Immediate recall, implicit	0.64	0.27	0.33
							Immediate recall, explicit	0.56	0.18	0.33
							Immediate recall, total	0.60	0.23	0.33
							Delayed recall (1 month), implicit	0.61	0.30	0.33
							Delayed recall (1 month), explicit	0.52	0.19	0.33
							Delayed recall (1 month), total	0.56	0.25	0.33
							Combined, implicit	0.64	0.20	0.33
							Combined, explicit	0.54	0.16	0.33
							Combined, total	0.59	0.18	0.33
Schwarz & Hamburger (2023b) *(Memory efects…)*^53^	Odor-cued navigation	Containers	Virtual reality	Unclear *(But explicit instruction to recall - participants had to decide direction based on landmark)*	Proportion of correct route decisions based on olfactory landmarks (larger = better)	25	Immediate recall	0.59	0.8	0.33
							Delayed recall (1 month)	0.53	0.75	0.33
							Combined	0.56	0.78	0.33
Szychowska, et al. (2025)^51^	Odor-place association	Olfactometer	Virtual reality	Yes	Distance from the correct location (smaller = better) *(Note: Values not available in the original manuscript were calculated from the raw data*,* e.g.: SD)*	25	Participants that first encoded location of odors (smells encoded first) T1: Immediate recall T2: 15-min delayed recall T3: 1-week delayed recall	6.57 10.70 13.07	3.47 4.97 5.71	19.06
						25	Participants that first encoded location of sounds (smells encoded second) T1: Immediate recall T2: 15-min delayed recall T3: 1-week delayed recall	5.84 6.37 10.61	3.37 3.44 3.70	19.06
						50	Combined T1: Immediate recall T2: 15-min delayed recall T3: 1-week delayed recall	6.21 8.54 11.84	3.41 4.76 4.92	19.06
Takahashi (2003)^36^ Study 1	Odor-place association	Paper	Walking between the rooms	Yes	Proportion correct for odor-room pairs	20	Participants with the instruction to memorize odor location	0.61	0.24	0.5
					Proportion incorrect			0.39	0.24	0.5
					d prime			0.56	1.23	0
				No *(but explicit instruction to recall)*	Proportion correct for odor-room pairs	20	Participants without the instruction to memorize odor location	0.58	0.24	0.5
					Proportion incorrect			0.43	0.24	0.5
					d prime			0.39	1.12	0
Takahashi (2003)^36^ Study 2	Odor-place association	Paper	Walking between the rooms	Yes	Proportion correct for odor-room pairs	20	Participants with the instruction to memorize odor location	0.71	0.16	0.5
					Proportion incorrect			0.29	0.16	0.5
					d prime			0.95	0.94	0
				No *(but explicit instruction to recall)*	Proportion correct for odor-room pairs	20	Participants without the instruction to memorize odor location	0.56	0.18	0.5
					Proportion incorrect			0.44	0.18	0.5
					d prime			0.29	0.90	0

In cases when participants were divided into groups performing spatial memory tasks for different sensory modalities, only groups from olfactory tasks are shown. Column “Explicit memorization instruction” indicates whether the instructions to participants included memorizing odor-place associations and information about having spatial memory tested; “Outcome measure” describes how the olfactory spatial memory was measured, and whether high or low score indicates better memory; “N” refers to the number of participants, “M” = the mean score, “SD” = standard deviation, and “Reported chance level” is the chance level as reported in the articles (NA = not reported). For the studies marked with an asterisk (*) – data was estimated from figures.

#### Differences between modalities

##### Qualitative synthesis

**Fig. 4:**
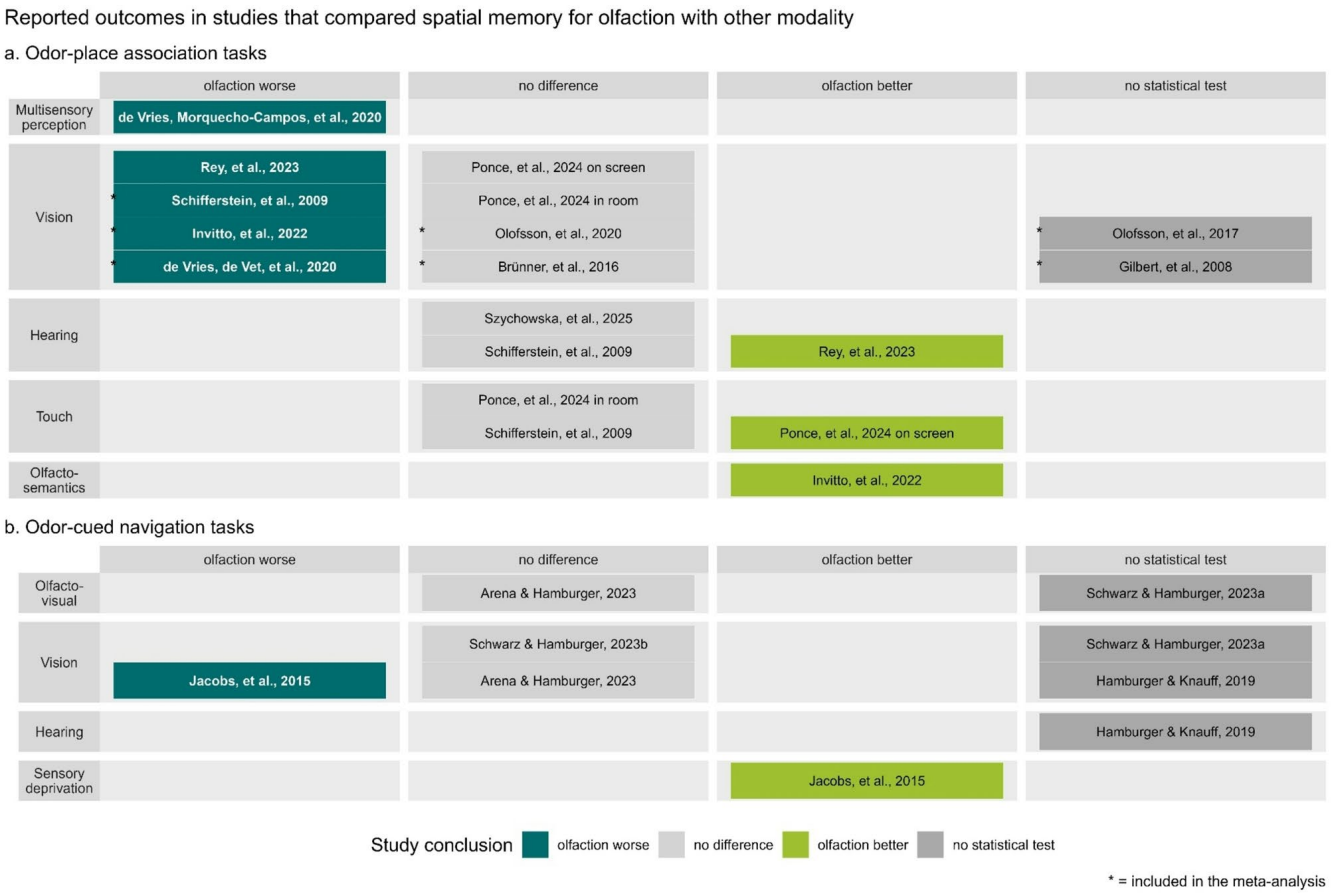
Reported outcomes of a comparison between spatial memory tasks in olfactory and other sensory conditions. Studies are separated by the type of memory task (a. odor-place association task, and b. odor-cued navigation task), the sensory modality to which results of the olfactory task were compared (in boxes of rows), and the reported outcome (columns). Color coding reflects the conclusion reported in the study supported by statistical analysis and unclear outcome when no statistical test was done. Note that some studies compared performance across multiple modalities, and these occur several times in the figure.

Figure 4 shows results of spatial memory performance comparison between olfaction and other conditions, as reported in the individual articles. Table 3 shows data extracted from the studies that compared olfactory spatial memory with visual spatial memory, and Table 4 shows data from the studies that compared olfactory spatial memory with other sensory conditions. Note that Hamburger and Knauff^55^ did not test other sensory conditions, but in the discussion section they briefly compare their results on olfactory spatial memory performance with results from a previous study that used identical design to test visual and auditory spatial memory performance^57^. Similarly, Ponce et al.^42^ statistically compared the results from the presented (olfactory) study with results from their previous article that investigated visual and tactile spatial memory^58^. Data from these comparisons are shown in Fig. 4 and in Tables 3 and 4, but are not included in the meta-analysis. Four studies presented results for the participants divided into two groups: athletes and non-athletes^39^, those training visual memory and olfactory memory^40^, younger and older adults^41^, and participants that first encoded location of odors and then sounds and those that first encoded location of sounds and then odors^51^. Data from the individual groups, and combined results are presented in Tables 3 and 4, but Fig. 4 and the meta-analysis only include the combined data.

Some studies reported multiple outcomes, for simplicity we present only selected ones (i.e., the ones that are most common across all studies). Specifically, for the study by Ponce et al.^42^, Tables 3 and 4, and Fig. 4 shows results for the number of correct object-place associations in both: the in-room recall, and on-screen recall. For the study by Rey et al.^46^, Table 3; Fig. 4 show the results for odor-context-place association. For the study by Szychowska et al.^51^, Table 4; Fig. 4 show the result for the distance error (distance from the correct location).

Furthermore, some studies tested spatial memory on several occasions. For the studies by Schwarz and Hamburger (*Memory effects…*)^53^ and Arena and Hamburger^52^, Tables 3 and 4 show the results for immediate and delayed recall, and combined across two times of testing, and Fig. 4 shows the reported results of ANOVAs. Finally, for another study by Schwarz and Hamburger (*Implicit versus…*)^54^, Tables 3 and 4 show the results for immediate and delayed recall and across two times of testing, combined across implicit and explicit trials. For the study by Szychowska et al.^51^, Table 4 shows the results for immediate, 15-minutes delayed, and 1-week delayed recall, and Fig. 4 shows results for the immediate recall as reported in the original article. For more details, see Supplementary material (“Supplement_SR_olfactory_spatial_memory.xlsx”).

**Table 3:** Olfaction versus vision: summary of results, including subject design, type of an outcome measure, number of participants (*N*), mean (*M*) and standard deviation (*SD*) of the outcome measure in each condition, and difference between the olfactory and visual condition (Mean diff). Observations in bold are included in the meta-analysis. For the studies marked with an asterisk (*) – data was estimated from figures.

Study authors (year) and condition/group	Subject design	Outcome measure	Olfaction			Vision			Mean diff (Olf − Vis)
			*N*	M	SD	*N*	M	SD	
Arena & Hamburger (2023)^52^ Immediate recall	between	Proportion of correct route decisions based on landmarks (larger = better)	16	0.64	0.16	18	0.70	0.12	−0.06
Arena & Hamburger (2023)^52^ Delayed recall	between	Proportion of correct route decisions based on landmarks (larger = better)	16	0.55	0.15	18	0.56	0.13	−0.01
Arena & Hamburger (2023)^52^ Combined	between	Proportion of correct route decisions based on landmarks (larger = better)	16	0.60	0.16	18	0.63	0.14	−0.03
*Brünner et al. (2016)^45^	**within**	**Number of correct odor-place associations** **(max 8**,** larger = better)**	**16**	**3.16**	**1.75**	**16**	**3.20**	**2.01**	**−0.04**
**de Vries**,** de Vet**,** et al. (2020)**^20^	**between**	**Distance error from correct location [pixels]** **(smaller = better)** *(Note: Means and SD calculated from the supplementary data files.)*	**88**	**135.49**	**46.94**	**88**	**129.71**	**81.73**	**5.78**
*Gilbert et al. (2008)^41^ young adults	between	Number of errors (smaller = better)	13	0.98	1.16	13	0.32	0.64	0.66
*Gilbert et al. (2008)^41^ older adults	between	Number of errors (smaller = better)	13	3.00	2.16	13	0.5	1.1	2.5
**Gilbert et al. (2008)**^41^ **combined**	**between**	**Number of errors ** **(smaller = better)**	**26**	**1.99**	**1.74**	**26**	**0.41**	**0.89**	**1.58**
Hamburger & Knauff (2019)^55^ + Karimpur & Hamburger (2016)^57^	between	Proportion of correct route decision based on olfactory landmarks (larger = better)	24	0.64	0.19	12	0.66	0.15	−0.02
Invitto et al. (2022)^39^ non-athletes	within	Span in Corsi Test (larger = better)	153	4.61	2.19	153	5.57	1.06	−0.96
Invitto et al. (2022)^39^ athletes	within	Span in Corsi Test (larger = better)	83	3.37	2.10	83	5.30	0.85	−1.93
**Invitto et al. (2022)**^39^ **combined**	**within**	**Span in Corsi Test ** **(larger = better)**	**236**	**4.17**	**2.16**	**236**	**5.48**	**0.99**	**−1.31**
Jacobs et al. (2015)^56^	within	Distance error from correct location [cm] (smaller = better)	83	289	146	83	12.3	12.3	276.7
*Olofsson et al. (2017)^48^	**within**	**Proportion of hits in the memory game ** **(larger = better)**	**13**	**0.42**	**0.08**	**13**	**0.47**	**0.09**	**−0.05**
Olofsson et al. (2020)^40^ olfactory training	within	N trials needed to complete the game (smaller = better)	41	38.1	10.8	41	33.8	10.3	4.30
Olofsson et al. (2020)^40^ visual training	within	N trials needed to complete the game (smaller = better)	31	40.1	10.5	31	37.9	8.80	2.20
**Olofsson et al. (2020)**^40^ **Combined**	**within**	**N trials needed to complete the game ** **(smaller = better)**	**72**	**39**	**10.7**	**72**	**35.6**	**9.6**	**3.396**
Ponce et al. (2024)^42^ + Munoz-Montoya et al. (2021)^58^ In room	between	Number of correct object-place associations (max 8, larger = better) *(Note: Means and SD for in-room recall are provided by the authors after email exchange*,* in parenthesis are Medians and IQR values from the article)*	25	7.44 (8)	1.04 (1)	47	7.36 (8)	0.92 (1)	0.08
Ponce et al. (2024)^42^ + Munoz-Montoya et al. (2021)^58^ On screen	between	Number of correct object-place associations (max 8, larger = better) *(Note: Means and SD for vision and touch in on-screen recall are calculated from the supplementary material available online)*	25	NA (6)	NA (3)	47	6.21 (6)	1.5 (1.5)	NA
Rey et al. (2023)^46^	within	Probability to reach the full episode after cue recognition (larger = better)	54	26.0	2.9	54	48.2	4.5	−22.2
**Schifferstein et al. (2009)**^21^	**between**	**Proportion of correct relocations ** **(larger = better)**	**20**	**0.22**	**0.16**	**20**	**0.30**	**0.17**	**−0.08**
Schwarz & Hamburger (2023a)^54^ Immediate recall	between	Proportion of correct route decisions based on landmarks (larger = better)	16	0.60	0.23	18	0.62	0.36	−0.02
Schwarz & Hamburger (2023a)^54^ Delayed recall	between	Proportion of correct route decisions based on landmarks (larger = better) *(Note: Mean score for Vision (0.55) was calculated as a pooled mean from implicit and explicit averages in the delayed recall written in the text*,* and the value for the implicit average given in the text differs from the one shown on the figure in the article.)*	16	0.56	0.25	18	0.55	0.30	0.01
Schwarz & Hamburger (2023a)^54^ Combined	between	Proportion of correct route decisions based on landmarks (larger = better)	16	0.59	0.18	18	0.56	0.36	0.03
Schwarz & Hamburger (2023b)^53^ Immediate recall	between	Proportion of correct route decisions based on landmarks (larger = better)	25	0.59	0.80	27	0.66	0.78	−0.07
Schwarz & Hamburger (2023b)^53^ Delayed recall	between	Proportion of correct route decisions based on landmarks (larger = better)	25	0.53	0.75	27	0.55	0.78	−0.02
Schwarz & Hamburger (2023b)^53^ Combined	between	Proportion of correct route decisions based on landmarks (larger = better)	25	0.56	0.78	27	0.60	0.78	−0.04

**Table 4:** Olfaction versus other sensory modalities: summary of results, including name of the compared sensory modality *(note: multisensory perception = vision + taste + olfaction)*, subject design, type of an outcome measure, number of participants (N), mean (M) and standard deviation (SD) of the outcome measure in each condition, and difference between the conditions (Mean diff). For the studies marked with an asterisk (*) – data was estimated from figures.

Article	Compared modality	Subject design	Outcome measure	Olfaction			Compared modality			Mean diff (Olf − Oth)
				*N*	M	SD	*N*	M	SD	
de Vries, Morquecho-Campos, et al. (2020)^43^	Multisensory perception	between	Proportion of correct relocations (larger = better)	254	0.36	0.24	258	0.58	0.29	−0.22
Arena & Hamburger (2023)^52^ Immediate recall	Olfacto-visual	between	Proportion of correct route decisions based on landmarks (larger = better)	16	0.64	0.16	19	0.68	0.14	−0.04
Arena & Hamburger (2023)^52^ Delayed recall	Olfacto-visual	between	Proportion of correct route decisions based on landmarks (larger = better)	16	0.55	0.15	18	0.49	0.12	0.06
Arena & Hamburger (2023)^52^ Combined	Olfacto-visual	between	Proportion of correct route decisions based on landmarks (larger = better)	16	0.60	0.16	18	0.59	0.16	0.01
Schwarz & Hamburger (2023a)^54^ Immediate recall	Olfacto-visual	between	Proportion of correct route decisions based on landmarks (larger = better)	16	0.60	0.23	18	0.75	0.30	−0.15
Schwarz & Hamburger (2023a)^54^ Delayed recall	Olfacto-visual	between	Proportion of correct route decisions based on landmarks (larger = better)	16	0.56	0.25	18	0.49	0.42	0.07
Schwarz & Hamburger (2023a)^54^ Combined	Olfacto-visual	between	Proportion of correct route decisions based on landmarks (larger = better)	16	0.59	0.18	18	0.67	0.28	−0.12
Hamburger & Knauff (2019)^55^ + Karimpur & Hamburger (2016)^57^	Hearing	between	Proportion of correct route decision based on olfactory landmarks (larger = better)	24	0.64	0.19	11	0.71	0.12	−0.07
Rey et al. (2023)^46^	Hearing	within	Probability to reach the full episode after cue recognition (larger = better)	54	26.0	2.9	54	14.8	2.00	11.20
Schifferstein et al. (2009)^21^	Hearing	between	Proportion of correct relocations (larger = better)	20	0.22	0.16	20	0.23	0.16	−0.01
Szychowska et al. (2025)^51^ Immediate recall	Hearing	within	Distance from the correct location (smaller = better)	50	6.21	3.41	50	6.08	3.65	0.13
Szychowska et al. (2025)^51^ 15-minutes delayed recall	Hearing	within	Distance from the correct location (smaller = better)	50	8.54	4.76	50	9.43	5.28	−0.89
Szychowska et al. (2025)^51^ 1-week delayed recall	Hearing	within	Distance from the correct location (smaller = better)	50	11.84	4.92	50	12.02	5.57	−0.18
Ponce et al. (2024)^42^ + Munoz-Montoya et al. (2021)^58^ In room	Touch	between	Number of correct object-place associations (max 8, larger = better) *(Note: Means and SD for in-room recall are provided by the authors after email exchange*,* in parenthesis are Medians and IQR values from the article)*	25	7.44 (8)	1.04 (1)	47	7.11 (7)	0.96 (1)	0.33
Ponce et al. (2024)^42^ + Munoz-Montoya et al. (2021)^58^ On screen	Touch	between	Number of correct object-place associations (max 8, larger = better) *(Note: Means and SD for vision and touch in on-screen recall are calculated from the supplementary material available online)*	25	NA (6)	NA (3)	47	4.34 (4)	1.96 (1.5)	NA
Schifferstein et al. (2009)^21^	Touch	between	Proportion of correct relocations (larger = better)	20	0.22	0.16	20	0.25	0.17	−0.03
Invitto et al. (2022)^39^ non-athletes	Olfacto- semantics	within	Span in Corsi Test (larger = better)	153	4.61	2.19	153	3.22	3.06	1.39
Invitto et al. (2022)^39^ athletes	Olfacto- semantics	within	Span in Corsi Test (larger = better)	83	3.37	2.10	83	0.51	1.71	2.86
Invitto et al. (2022)^39^ combined	Olfacto- semantics	within	Span in Corsi Test (larger = better)	236	4.17	2.16	236	2.27	2.67	1.90
Jacobs et al. (2015)^56^	Sensory deprivation	within	Distance error from correct location (in cm, smaller = better)	83	289	146	83	361	153	−72

As shown in Fig. 4 (and supported by data in Tables 3 and 4), the results of the empirical studies suggest that olfactory spatial memory measured in terms of odor-cued navigation is either worse than, or does not differ from, visual spatial memory^52,53,56^, does not differ from olfacto-visual spatial memory^52^, and is better than navigation in a sensory-deprived condition^56^. For spatial memory measured in terms of odor-place association, olfactory spatial memory seems to be worse than multisensory spatial memory^43^; Better or the same as auditory spatial memory^21,46,51^; Better or the same as tactile spatial memory^21,42^; And better than spatial memory for olfacto-semantic objects^39^. Most studies compared odor-place association to visual object-place association, and as shown in Fig. 4; Table 3 olfactory spatial memory is worse or similar to its visual counterpart^20,21,39,40,42,45,46^. To quantitatively evaluate this particular comparison, we performed meta-analysis on the available data.

##### Meta-analysis: olfaction versus vision

We identified 12 articles where olfactory and visual spatial memory performances were compared within the same study^20,21,39–41,45,46,48,52–54,56^. Four studies followed a design that resembled an odor-cued navigation^52–54,56^, and the other studies used different versions of odor-place association tasks. Furthermore, one study tested three-way associations (odor-context-place) rather than simple odor-place associations^46^. Because of methodological differences, we decided to not include the five deviating studies, leaving seven articles in the meta-analysis.

Three studies tested spatial memory in two separate groups: Gilbert et al.^41^ tested young and older adults; Invitto et al.^39^ tested athletes and non-athletes; and Olofsson et al.^40^ divided participants into groups designated for olfactory or visual training. In these three studies, we estimated means and SDs pooled across different groups, and submitted combined values to the meta-analysis. Additionally, in one study participants memorized odor-place associations in two distinct olfactory and two distinct visual conditions^45^. We combined the results within sensory modalities using formulas for pooled means and SDs and used the combined values in the meta-analysis. Finally, two studies tested the performance before and after training^40,48^. From these studies, performance before training was included in the meta-analysis.

Notably, some studies reported outcomes, in which smaller scores indicated better performance and others reported outcomes, in which larger scores indicated better performance. The scores were re-coded for the meta-analysis to ensure that the difference score represents the correct direction of the effect. Finally, even though only three studies used a between-subject design while the rest used a within-subject design, we decided to employ a conservative approach and combine the evidence by treating all the seven studies as having a between-subject design.

**Fig. 5:**
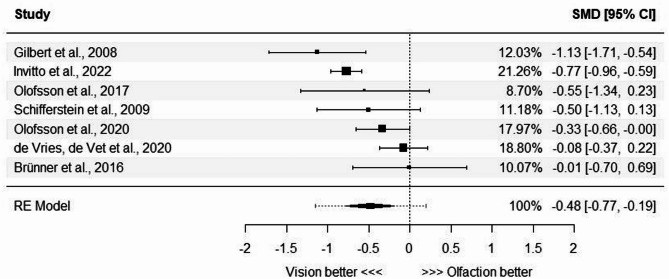
Effect sizes of the meta-analysis for standardized mean differences (SMD) in performance between olfactory and visual spatial memory (odor/object-place associations). Global effect size of the meta-analysis is represented as a diamond at the bottom, together with 95% prediction intervals calculated because of the heterogeneity. Error bars represent 95% confidence intervals for standardized mean differences from each study.

A total of *k* = 7 studies were included in the analysis (in bold in Table 3; Fig. 5)^20,21,39–41,45,48^. The observed standardized mean differences ranged from − 1.13 to − 0.01, with all estimates being negative (100%). The estimated average standardized mean difference (i.e., Glass’s estimator obtained with metafor::escalc()) based on the random-effects model was $\:\widehat{\mu\:}$ = −0.48, *95% CI* [− 0.77, − 0.19] and the average outcome differed significantly from zero (*z* = − 3.22, *p* = 0.0013), suggesting that performance is statistically worse in olfactory than visual spatial memory tasks (Fig. 5). According to the Q-test, the true outcomes appear to be heterogeneous (*Q(6)* = 23.03, *p* = 0.0008, *tau*^*2*^ = 0.095, *I*^*2*^ = 71.09%) with the I^2^ score implying moderate-to-high heterogeneity (i.e., 50% < *I*^*2*^ < 75%)^59^. The 95% prediction interval for the true outcomes is between − 1.15 and 0.19, meaning that although the average outcome is estimated to be negative, in some studies the true outcome may in fact be positive. In other words, on average, people tend to perform worse in the olfactory spatial memory tasks rather than their visual counterparts, but in some individual studies the results could show comparable or even slightly better performance in olfactory tasks.

**Fig. 6:**
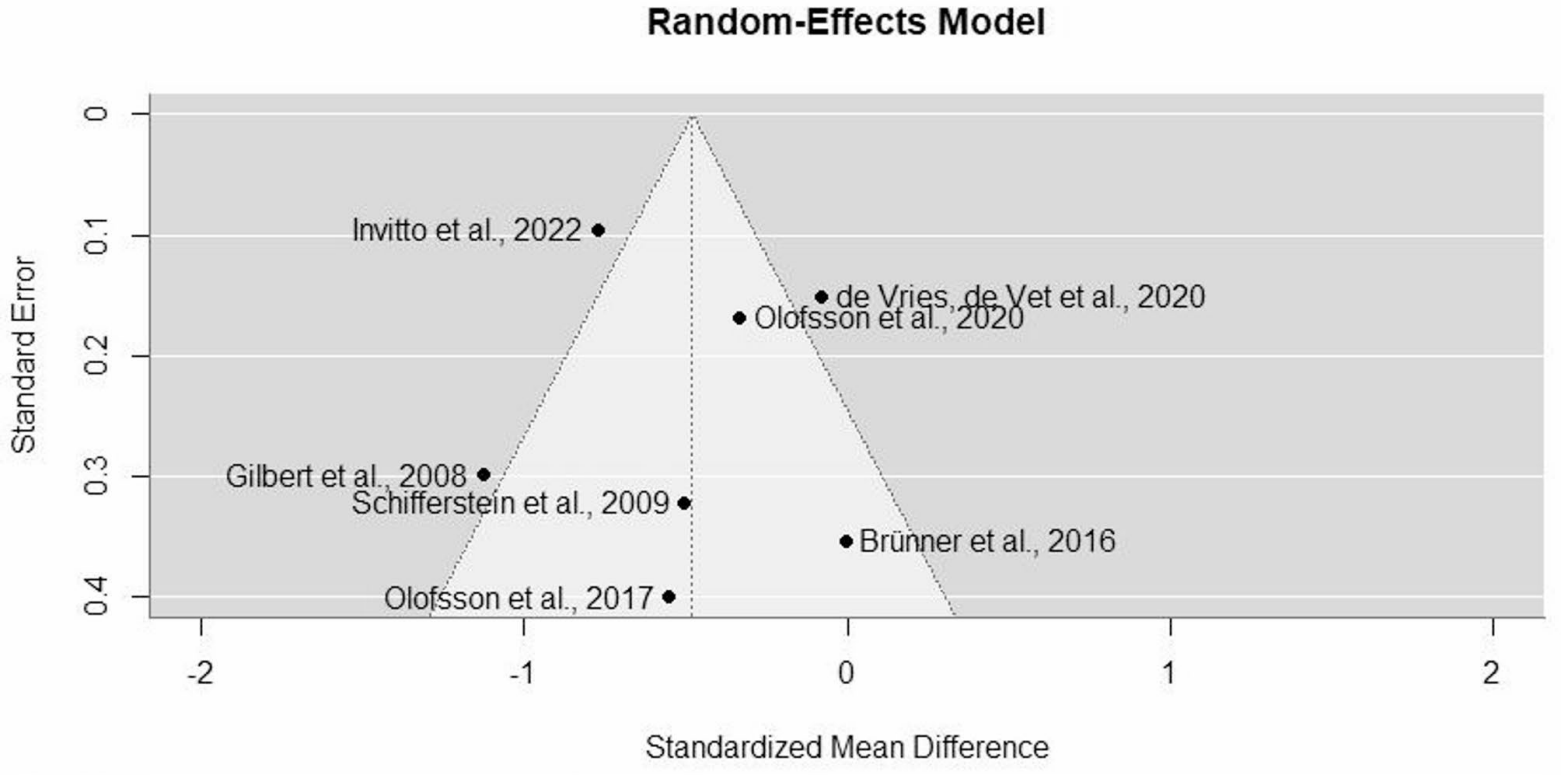
Visual inspection of publication bias in the form of a funnel plot. Each dot represents a study included in the meta-analysis. The y-axis represents study precision as measured by the standard error; the x-axis shows standardized mean difference in performance between olfactory and visual spatial memory (object-place associations) in each study.

An examination of the studentized residuals revealed that values for studies varied between − 1.72 and 1.55, which suggests that there were no outliers in the context of this model. According to Cook’s distances, none of the studies could be considered to be overly influential (all *Cook’s d* < 0.45). Finally, neither the rank correlation (*Kendall’s tau* = 0.05, *p* = 1.0) nor the regression test (*z* = −0.008, *p* = 0.99) indicated any funnel plot asymmetry (Fig. 6).

## Discussion

We conducted a systematic review and meta-analysis of results regarding human olfactory spatial memory abilities, and we compared performance in spatial memory tasks between olfactory and visual sensory modalities. Spatial memory is often defined as the cognitive ability to store and recall spatial information about the arrangement of objects or routes^2^. Encoding object locations might be operating through different processes than encoding of specific routes and pathways (either because of incorporating sequential information into route encoding, or using stimulus-action associations rather than spatial maps^6^, and previous reviews and meta-analyses on spatial memory present these tasks separately^2,38^. In this systematic review, we found studies that tested spatial memory for both layouts (odor-place associations) and routes (odor-cued navigation). In both cases, studies exhibited large variation in types of tasks used to evaluate spatial memory abilities, in odor-delivery, and in spatio-contextual environment. Notably, this review did not include studies where spatial memory was tested as a fully implicit phenomenon.

### Odors as landmarks for creating cognitive maps

Humans can navigate mazes and find their way using odor cues when visual cues are ambiguous^18,52–56^, in line with a recent review and theoretical discussion on odor-cued navigation in humans^8^. Odors can therefore serve as landmarks^18,52–55^ and might be a basis for creating cognitive maps in both higher cognitive areas and primary sensory areas of the human brain^18^. These findings align with rodent models, in which olfactory cues in spatial tasks indeed contribute to formation of cognitive maps in cognitive areas^5,7,60^, and in primary olfactory cortex^6^. Furthermore, cognitive maps based on olfactory landmarks might be resistant to time-related decay due to high emotional salience of odors, and due to implicit rather than explicit encoding of odors, which could make the maps resistant to interference^52–54^.

### Possible distinct Spatial memory for food-related odors

Humans are able to memorize locations of smells and seem to perform particularly well for locations of odors related to food^44^. Furthermore, locations of odors associated with high-calorie content might be memorized better than those associated with low-calorie content^20,43^. This high-calorie bias was also consistently shown in subsequent food and taste studies by the same research team^61,62^. A recent meta-analysis showed that food-related odors result in a different brain activation than non-food odors in areas typically related to spatial memory and navigation^63^. In particular, food odors showed stronger activations in the entorhinal cortex than non-food odors, which together with the results of this review, might point to a better integration of food odors with the memory network. However, the effect of hunger on these results remains unclear, as only one study clearly stated that results were unaffected by the hunger rating^43^. Altogether, the evidence for distinct spatial memory for food-related odors is still limited and requires further investigation, with careful consideration of the potential hunger effects.

### Singular memory process for odor-recognition and odor-context-place associations

When odor is associated with a visual context (e.g., specific landscape) plus a specific location within that context, once the odor is correctly recognized, humans tend to remember the entire odor-context-place association^37,47,49,50^, and tend to respond quicker when they are able to recall the entire odor-context-place association rather than just odor-context or odor-place associations^46,49^. Throughout several studies, the authors conclude that such results might indicate that odor-recognition and odor-context-place association stem from a singular memory process, even though no neurological recordings were obtained. Nevertheless, previous studies on both rodents and humans suggested that overlapping brain networks might underlie recognition memory and spatial memory^64–66^, which could potentially be in line with the presented behavioral findings. Similar behavioral results were found for face-context-place associations, where the response time was shortest when the entire association was correctly recalled, but not for music-context-place associations^46^. Therefore, the generalizability of this effect to other modalities remains unclear.

### Training and repetition improve olfactory Spatial memory

Finally, several studies showed that spatial memory for smells can be improved with repetition during one day or with training across several days^18,40,44,48^. However, training effects might not be present without explicit instruction to memorize odor-place associations, or for more complex odor-place-context associations^37^. Interestingly, the effects of training in olfactory spatial memory might transfer to other domains, such as other olfactory tasks, or even visual spatial memory^40,48^. However, although olfactory training is receiving increased interest due to its presumed sensory, emotional, and cognitive benefits^67^, more evidence is needed to validate the proposed olfaction-based memory transfer effects.

### Comparing olfactory Spatial memory with other modalities

Results of the qualitative synthesis and meta-analysis that compared olfactory and visual spatial memory performance showed that performance in olfactory tasks was significantly worse than performance in their visual counterparts. This finding is not in line with the proposed unique role of olfaction as having evolved together with spatial memory systems to aid spatial navigation^14,22,23,68^. Instead, the unique connectivity between olfactory primary areas and hippocampus and entorhinal cortex, the brain’s centers for spatial memory and navigation^9^ may not give olfaction a behavioral advantage in terms of the spatial memory abilities.

One possible explanation might be related to the finding that performance in spatial memory tasks tends to be better for objects that can be easily identified and labeled^69^. In the reviewed studies, even though participants were sometimes tested on odor recognition or identification before or after the memory task, participants were rarely asked to label the odor spontaneously during the memory task, adding a degree of uncertainty to whether the odors were correctly identified or labeled during memory encoding and recall. Specifically in the studies included in the meta-analysis, the identifiability of olfactory stimuli was often not reported by the authors, except one study that aimed for the odors to be difficult to identify^21^ (see Supplement Table 6 in Supplement_1_tables_and_figures.docx, for quick preview of the meta-analysis studies, and Supplement_SR_olfactory_spatial_memory.xlsx for details on all reviewed studies). Critically, even familiar odors are difficult to label, to the level at which olfaction has been named “a muted sense”^70^. Therefore, it is possible that any advantage that the olfactory system might have because of the direct and strong neural connectivity to the aforementioned cognitive centers might be counterbalanced by the semantic difficulties in labeling the odors in the odor-place association tasks.

However, even though most reviewed studies lack the information about participants’ ability to spontaneously identify or label the odors because it was not tested and/or not reported, and some studies used odors that are supposed to be difficult to identify, we cannot fully exclude that the participants were labelling the odors. Notably, olfaction might be highly affected by verbal information^71,72^, and adding additional semantic information to odors could affect the validity of the task^52^. Adding semantic information to odors has the potential to strengthen the memory trace^50,73–77^, or even supersede it^73^ to the extent where the task may potentially change from olfactory to semantic spatial memory. One of the reviewed studies tested whether there was a difference in spatial memory performance between easy-to-identify and hard-to-identify odors (divided with a median split based on a pilot study). Results suggested no difference in spatial memory performance between the two groups^55^.

Another explanation could be that associating objects with specific locations is a task simply more suitable for vision, which provides an unparalleled mapping of spatial coordinates and in regards to spatial memory is superior also to auditory and tactile spatial abilities^58,78–80^. Instead, olfaction might have evolved to aid a different type of spatial behavior, which may be not accessible with the visual sense. For example, rather than supporting memory for odors locations, its spatial purpose could be to complement the visual information through the ability to perceive and track odor gradients^56^, or to reorient visuospatial attention and guide spatial behavior in terms of approaching locations with pleasant smells and avoiding unpleasant ones^81,82^.

Finally, it is possible that olfaction is primarily used implicitly in guiding spatial behavior^8,54^, or even that olfaction might simply not be a sense relevant for any spatial behavior in humans because all spatial problems could be much quicker and easier solved with vision, and odors are unreliable as they might be easily affected by weather conditions (e.g., wind)^83^. Notably, there seem to be two distinct approaches to the relationship between olfaction and spatial cognition. One with olfaction treated as a sense relevant for spatial behavior and cognition (either explicitly or implicitly)^8,15,22,23,68^, and the other where olfaction has no function in human spatial behavior and cognition because of lack of ecological importance^27,28,83^. Still, the unique structural and functional connectivity between olfaction and spatial centers in our brain exists and researchers should try to understand the purpose of this close integration.

Results from a few studies showed that olfactory spatial memory is similar or better than auditory, tactile, olfacto-visual, olfacto-semantic, and sensory-deprived spatial memory^21,39,42,46,51,52,55,56^. In fact, it is only studies that compare olfaction with vision or multisensory condition that reported worse performance for olfaction^21,39,43,46,56^. Therefore, while vision is the primary sense humans use to memorize locations of objects in space, olfaction might be following just behind, surpassing other sensory modalities. However, the evidence for these outcomes is limited and should be investigated further. Altogether, our results clearly indicate that human spatial memory does not interact similarly with all the senses, and therefore generalizing empirical findings across sensory modalities should be avoided^8,83^.

### Implicit versus explicit instructions

Most of the reviewed studies used explicit memorization instruction. However, a series of studies on implicit odor memory showed that rooms are more often associated with a specific odor only if that odor has not been recognized as previously encountered in the room^27,28^. In line with that, results of one study reviewed here showed better performance in odor-cued navigation spatial memory task for odors that were not correctly recognized as landmarks, as compared with those that were correctly recognized^54^. The authors argue that these results indicate that odors are better used in guiding spatial behavior when they are processed implicitly rather than explicitly, in contrast to visual stimuli. In contrast, two studies (in one reviewed article) that tested odor-room association memory in two groups of participants*—*one group with explicit memorization instruction, and the other without*—*showed better memory performance in the explicit memorization group^36^. Therefore, future studies should carefully consider the effects of explicit instructions on spatial memory performance when designing experiments.

### Limitations and future directions

The present systematic review and meta-analysis has some limitations. First, the results of the meta-analysis showed moderate-to-high heterogeneity which is common amongst meta-analyses with a small number of studies (k = 7 in the present meta-analysis)^59^. Additionally, even though we included only the studies that most likely measured the same cognitive process (object-place association), there was a large variation across studies in the methods and materials used in the experimental protocols (see Fig. 3 for all studies and Supplement Fig. 2 for summary of methods only in studies included in the meta-analysis), which could have contributed to the size of heterogeneity. Notably, not all the studies employed an odor recognition test to investigate whether participants were able to correctly recognize or identify the stimuli (see “Supplement_SR_olfactory_spatial_memory.xlsx”), and none of the studies tested whether the odors could be accurately labeled spontaneously during the memory task. Furthermore, the calculated prediction interval overlapped with 0, suggesting that for individual studies, the outcome might show similar performance between the two modalities or even better performance in the olfactory, rather than the visual task. Nevertheless, the absence of outliers and influential studies, along with the lack of funnel plot asymmetry, suggests robustness and reliability in the overall conclusion of inferior olfactory, relative to visual, spatial memory performance.

Another limitation is that the second largest difference between vision and olfaction was found in a study that scored lowest on the quality risk evaluation, and used a slightly different task to evaluate visual and olfactory spatial abilities^39^. Therefore, it might have compared outcomes of different cognitive processes, rather than the same cognitive process in different modalities. Furthermore, a study with the largest difference between modalities^41^, tested both younger and older (cognitively healthy) populations. In that study, the large difference between olfactory and visual performance was driven by the older population, even though the older population was cognitively healthy and neither odor recognition nor place recognition was impaired in older compared to younger group. Therefore, it is not unlikely that the heterogeneity and the obtained effect size are driven by these two studies.

Our review revealed an extensive variety of methodologies used in the reviewed scientific articles, including differences in odor delivery (e.g., presentation through an olfactometer, containers, sniffin’ sticks) and in visuo-spatial context (e.g., walking in a room, sitting at a table with marked locations, looking at the computer screen). This might be because of the difficulty to design a reliable and ecologically valid experimental protocol to study spatial organization of smells or odor-based route navigation. We believe that unifying or standardizing procedures to evaluate olfactory spatial memory is an important step in future research. One way to reach that goal could be incorporating virtual reality (VR) in cognitive research, as in some recent studies presented in this systematic review^51–54^. The VR provides a highly controllable and reproducible, yet more natural and interactive testing paradigm^84^. Notably, many reviewed studies used experimental paradigms where participants were sitting at the table or beside the computer screen, which seems artificial in the context of spatial navigation, as it deprives participants of movement and proprioceptive information commonly available in real life. A recent study showed better spatial memory performance in a condition where participants could move and actively explore the environment in augmented reality (AR), than when participants were performing the spatial memory task in the VR but while sitting, emphasizing the importance of movement during spatial memory encoding^85^. Therefore, ideally, future studies should use VR as a medium providing visuo-spatial context in a way that allows for incorporating proprioceptive and muscular signals into the spatial memory task. Such a solution would not only help standardizing the methods, but also increase the ecological validity of the task. A recent review on visual and visuo-spatial memory, which identified several issues in the way memory has been assessed over the years, also suggested exploring VR as a way to make the tests more reliable^86^. While using VR with visual objects is straightforward and requires no additional equipment, using VR with odors is more challenging. However, current technological developments begin to allow for VR-compatible, controllable, and reliable olfactory delivery^87–90^.

Furthermore, our search did not find any studies that investigated olfactory spatial memory in visually impaired individuals, identifying a critical knowledge gap. A couple of recent studies suggested that blind and sighted individuals perform similarly or better (i.e., congenitally blind) on odor lateralization and localization tasks^91,92^, and interviews with blind individuals indicated use of odors as, for example, points of reference in wayfinding^93^. Nevertheless, it remains unclear whether olfaction plays a different role in spatial memory for blind and for sighted, and it should be investigated.

Notably, while our review only included studies that allowed for a direct investigation of spatial memory through conscious recall, as one of the reviewers of this manuscript pointed out, memories can be not only formed, but also evaluated implicitly. For example, participants that performed tasks in scented rooms while being unaware of the odor tended to later evaluate the odor from the room as more fitting to the room than other presented odors^27,28^. This increase in the degree of fit could be interpreted as implicit memory for odor-room association and therefore—be an indirect measure of olfactory spatial memory. However, implicit memory was not the subject of the present manuscript, and for a literature on implicit olfaction in spatial behavior, we refer the reader to other publications^8,83^. Similarly, in addition to the results relevant for our purposes, some individual studies showcased interesting results that were not within the scope of this systematic review and are not discussed here. With regards to those, our review provides a comprehensive guide to the literature and might as such stimulate further research.

## Conclusions

This systematic review and meta-analysis provide insights into several aspects of olfactory spatial memory. First, combined results of behavioral and neuroimaging studies suggested that odors might have the potential to serve as the foundation for cognitive maps, which play a critical role in spatial memory and navigation tasks, in both higher cognitive areas and primary olfactory regions. These olfactory cognitive maps might be in particular resistant to long-term memory decay. Second, there might be a particular proficiency in memorizing locations of high-calorie food odors. Third, several studies suggested a potential unity or overlap in the memory-related processes that underlie odor recognition and olfactory spatial memory. Fourth, olfactory spatial memory skills might be improved with targeted training, which also showed potential transfer effects to other cognitive domains.

Nevertheless, human performance in olfactory spatial memory is inferior to visual spatial memory. Our results underscore the importance of moving away from generalizing results from one sense to another, as the performance in cognitive tasks, such as spatial memory, might not be comparable across sensory modalities. Addressing methodological variations and further exploring the neural mechanisms underlying olfactory spatial memory will be crucial for advancing this field of research.

## Supplementary Information

Below is the link to the electronic supplementary material.

Supplementary Material 1

Supplementary Material 2

Supplementary Material 3

Supplementary Material 4

Supplementary Material 5

Supplementary Material 6

## Data Availability

The study was not preregistered. All data for this project, along with relevant code and codebooks, have been made available on Open Science Framework and can be accessed at [https://osf.io/5y9vj/]^26^.
