# Supplementary material for: Olfactory spatial memory: a systematic review and meta-analysis
Source: Sci Rep. 2025 Nov 4;15:38469. doi: 10.1038/s41598-025-25503-5 (PMC12586518; doi:10.1038/s41598-025-25503-5)
Supplement: Supplementary file 1 — Supplementary Material 1 [file 41598_2025_25503_MOESM1_ESM.pdf]

Supplement 2 to the manuscript

Olfactory spatial memory: A systematic review and meta-analysis

ASReview application is a free-source tool, created to make the article screening process faster and more efficient (Van de Schoot et al., 2020; van de Schoot et al., 2021). It uses an active learning cycle, where machine learning algorithms are employed to continuously rearrange the order of articles for screening based on the reviewer's responses to previous articles, so that the relevant articles are selected as early in the process as possible. Notably, the application does not make the decision about the inclusion, it merely rearranges the order of screening. After automated title and abstract screening, we extracted analytical information from ASReview. Over 75% of the relevant articles were identified after only 14% (n = 86) of the articles were screened, and 100% of relevant articles were identified after only 54% (n = 340) of the articles were screened (Figure 2). This outcome supports the idea that the ASReview application is suitable and accurate in making article-screening process more efficient and recommend using it in the future reviews and meta-analyses, with a reasonable stopping criterion (for comparisons of stopping criteria, see: Oude Wolcherink et al., 2023; Scherhag & Burgard, 2023).

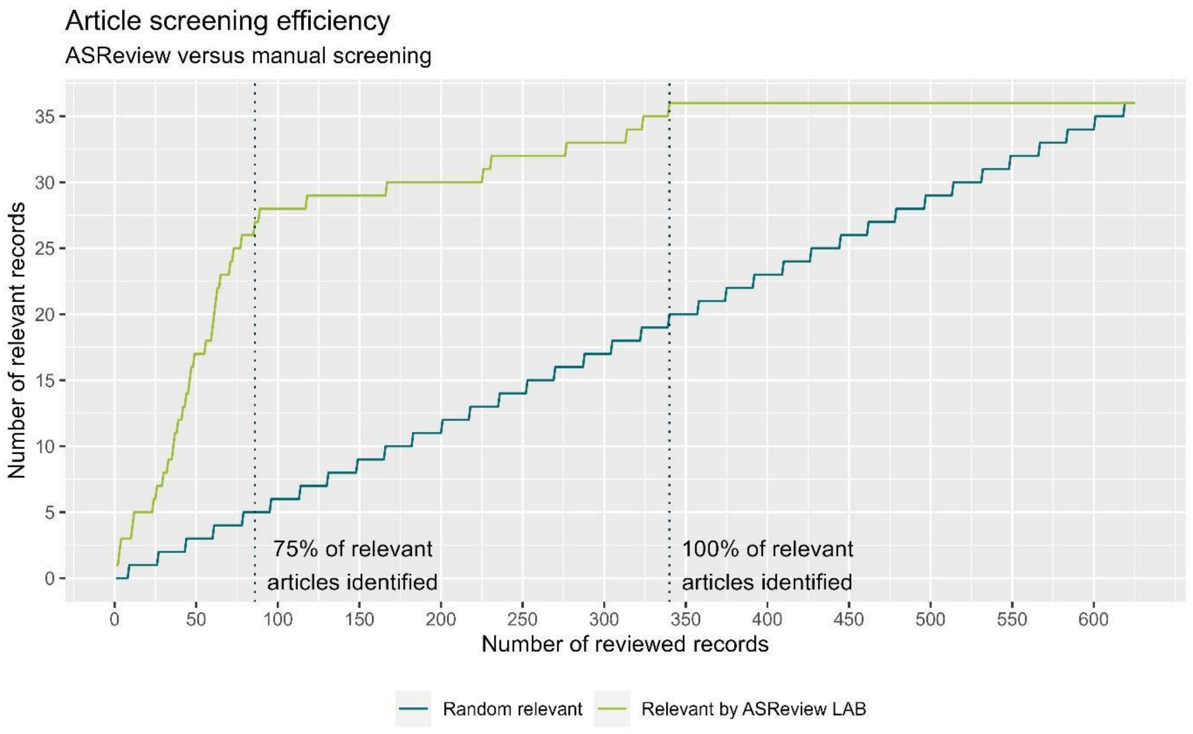

Supplement 2 Figure 1. Analytics from the ASReview application. Number of articles marked by the reviewer as relevant when utilizing machine learning capacities of ASReview (light green / light gray) as compared to number of relevant records if the articles were screened in a random order (turquoise

/ dark gray). Dotted lines highlight the number of records reviewed in order to find 75% or 100% of the relevant records using ASReview screening

References:

Oude Wolcherink, M. J., Pouwels, X. G. L. V., van Dijk, S. H. B., Doggen, C. J. M., & Koffijberg, H. (2023). Can artificial intelligence separate the wheat from the chaff in systematic reviews of health economic articles? *Expert Review of Pharmacoeconomics & Outcomes Research*, *23*(9), 1049–1056. https://doi.org/10.1080/14737167.2023.2234639

Scherhag, J., & Burgard, T. (2023, May). *Performance of Semi-Automated Screening Using Rayyan and ASReview: A Retrospective Analysis of Potential Work Reduction and Different Stopping Rules*. ZPID (Leibniz Institute for Psychology). https://doi.org/10.23668/psycharchives.12843

van de Schoot, R., de Bruin, J., Schram, R., Zahedi, P., de Boer, J., Weijdema, F., Kramer, B., Huijts, M., Hoogerwerf, M., Ferdinands, G., Harkema, A., Willemsen, J., Ma, Y., Fang, Q., Hindriks, S., Tummers, L., & Oberski, D. L. (2021). An open source machine learning framework for efficient and transparent systematic reviews. *Nature Machine Intelligence*, *3*(2), Article 2. https://doi.org/10.1038/s42256-020-00287-7

Van de Schoot, R., De Bruin, J., Schram, R., Zahedi, P., De Boer, J., Weijdema, F., Kramer, B., Huijts, M., Hoogerwerf, M., Ferdinands, G., Harkema, A., Willemsen, J., Ma, Y., Fang, Q., Tummers, L., & Oberski, D. (2020). *ASReview: Active learning for systematic reviews* [Computer software]. Zenodo. https://doi.org/10.5281/zenodo.4158671
