# Supplementary material for: Olfactory spatial memory: a systematic review and meta-analysis
Source: Sci Rep. 2025 Nov 4;15:38469. doi: 10.1038/s41598-025-25503-5 (PMC12586518; doi:10.1038/s41598-025-25503-5)
Supplement: Supplementary file 2 — Supplementary Material 2 [file 41598_2025_25503_MOESM2_ESM.pdf]

Qualitative report

Below, all included studies are summarized (see also Table 2 in the manuscript).

Brünner et al. (2015) asked participants to memorize odor-place pairs (modified Sniffin' sticks paired with a location on a computer screen), for either food-related or non-food related odors. Participants underwent two sessions of testing, one with insulin application and one with placebo. Because clinical applications are not of interest in this review, we only include data and results from the non-insulin testing session. In the three first recall occasions, participants received feedback about their performance, but in the fourth recall (10-minutes delayed), no feedback was provided. Results showed that performance improved with training during the initial recall tasks and slightly declined after time delay. In general, participants were better at memorizing location of food- rather than non-food-related odors, which might be explained by the evolutionary adaptation, and importance of remembering food locations for human fitness and survival.

Continuing the investigation of olfactory spatial memory, Brünner et al. (2016) asked participants to encode and recall location of odors or images in four different mazes, two mazes per sensory condition, while recording their brain activity with functional magnetic resonance imaging (fMRI). For the purpose of the meta-analysis, pooled means and SDs were calculated within each sensory modality from the behavioral results. Similar to a previous study from that research group, participants were tested on two occasions, one with insulin application and one with placebo, and in the current meta-analysis we only include the results from the placebo (control) session. Behavioral results showed no difference in performance between olfactory and visual conditions. Results of the fMRI analysis done across a subset of participants with and without insulin application showed brain activation of olfactory areas such as the orbitofrontal and piriform cortices as well as the amygdala and the hippocampal areas in the olfactory condition.

De Vries, de Vet, et al. (2020) asked participants to memorize odors or images coming from different food stalls (high- vs low- calorie content, and savory vs. sweet taste in 2x2 design) on a fictional campus map shown on the computer screen. Participants were not informed that their spatial memory will be tested. Then, participants were asked to recall locations of the stalls based on the presented odor. The authors found that participants performed slightly better in the visual than olfactory condition, and in both conditions, participants performed better for high rather than low-calorie food, and for savory rather than sweet tastes. These results were associated with the evolutionary adaptation, where remembering locations of high calorie, and savory (rich in protein) foods were beneficial for fitness and survival.

Similarly, de Vries, Morquecho-Campos, et al. (2020) asked participants to navigate in a maze-like room with different types of foods (high- vs low-calorie content, and savory vs. sweet taste) placed at different locations. Food was either represented by odors in the olfactory condition or was a real food in the multimodal condition. Participants were not informed that their spatial memory will be tested afterwards. In the recall test, participants were asked to indicate, on a computer screen, locations of foods that they encountered while walking around in the room. Participants performed better in the multimodal than olfactory condition, and in both conditions, participants performed better for high- rather than low-calorie content food. However, there were no differences in the sweet versus savory comparison (values not reported in the paper). These results were partially in line with two previous studies (Brünner et al., 2015; de Vries, de Vet, et al., 2020), supporting sensitivity of humans to fitness-relevant olfactory stimuli in a spatial situation.

Gilbert et al. (2008) asked younger and cognitively healthy older participants to memorize and recall location of particular odors or visual objects on a board in front of them. Six odors/objects were paired with six (out of 12) locations on the board, and presented one at a time. Results showed that older adults performed much worse (did more errors) than younger adults in the olfactory condition, but not in the visual condition. To control for aging effects on recognition memory, participants were tested with odor, object, and location recognition tests, and only those who did not exhibit any recognition impairments were included in the study. Therefore, the authors could isolate the decline specifically in olfactory associative memory in the older adults. The authors suggest that their results are caused by loss of ability to associate odors with spatial locations, which in turn may be caused by degeneration in hippocampal and parahippocampal regions involved in processing of memory for odors and space.

Goodrich-Hunsaker et al. (2009) tested amnesic patients and matched controls in an odor-place association task, where six odors were paired with six (out of 12) locations on a board in front of the participants, one odor at a time. Only results of the control sample are presented in Table 2. Results showed that amnesic patients were impaired in odor-place association and place-recognition tasks, but not in odor-recognition, compared to controls. Furthermore, most errors that both groups made were related to confusing odor-place pairs, so the odor was simply placed in a location paired with another odor, rather than in a location without an odor pair.

In a study by Hamburger and Knauff (2019), participants learned to use olfactory landmarks for navigating in a virtual maze-like environment (i.e., squareland, Hamburger & Knauff, 2011). Specifically, participants were asked to memorize the path through the maze only based on the olfactory landmarks (e.g., turn left at the vanilla odor). To ensure that participants made their route decisions based on olfactory landmarks and not on the spatial

sequential learning (memorizing the order of turns), participants also performed a control task, in which they were simply put at four randomly selected odor locations and asked to make a turn decision. Results showed that in both odor-based navigation tasks and control tasks, participants made around 64% correct route decisions (compared to 33% chance), indicating that participants indeed navigated the maze using olfactory landmarks. Based on these results, the authors argued that the human brain can use olfactory landmarks to build cognitive maps. Furthermore, the authors compared their current results with their previous study that investigated visual and auditory landmark-based navigation in an otherwise identical paradigm (Karimpur & Hamburger, 2016). Even though no statistical test was applied, the authors argued that humans perform almost equally well in navigation tasks using olfactory, auditory, or visual landmarks, which they view as a support for the notion that sensory modality of a landmark is irrelevant, as long as the landmark is perceptually salient (Caduff & Timpf, 2008). These conclusions were strengthened by replicating the results in a recent series of studies that utilized a similar task paradigm in virtual reality, where participants were asked to use olfactory, visual, or olfacto-visual landmarks to navigate through mazes (Arena & Hamburger, 2023; Schwarz & Hamburger, 2023b, 2023a). Results showed that participants made on average between 56% and 64% correct route decisions based on the olfactory landmarks, between 60% and 70% correct decisions based on the visual landmarks, and between 48% and 83% correct decisions based on the olfacto-visual landmarks. Therefore, the authors again argue for the ability of the cognitive maps to utilize information from different senses at a similar level. Interestingly, even though the results in navigation tasks did not differ between conditions, the recognition of stimuli was statistically worse for odors than for visual or olfactory-visual stimuli (Arena & Hamburger, 2023; Schwarz & Hamburger, 2023b). This finding led the authors to explore the effect of implicit and explicit stimuli on the performance. Results showed better spatial memory performance in the olfactory and olfacto-visual conditions for the stimuli that were not correctly recognized (implicit encoding), in contrast to the visual condition where performance in wayfinding was better for the stimuli that were also correctly recognized (explicit encoding) (Schwarz & Hamburger, 2023a). The authors discussed the ecological validity of these findings, arguing that in everyday life, humans indeed use odors in a somewhat unaware way, while visual information is used mainly explicitly. Additionally, in 3 studies, the researchers showed that performance in olfactory conditions did not significantly decline over time, in contrast to visual and olfactory-visual conditions (Arena & Hamburger, 2023; Schwarz & Hamburger, 2023b, 2023a). Authors attributed this finding to the idea that odors encoding is implicit and therefore processed on a lower level which might lead to less interference, and to the stronger emotional salience of odors.

Invitto et al. (2022) tested participants from athletic (students from the Motor Science degree) and non-athletic (students from the Psychology degree) study programs in three types of Corsi Block Tapping Test (CBTT): *Classical (visual) CBTT,* where participants were asked to reproduce the sequence in which blocks were touched by the experimenter, starting with a sequence of 2 blocks, and increasing up to 10 blocks (note, that in this condition participants had to remember the temporal order of presented blocks); *Olfactory CBTT*, where participants were presented with sequences of odors associated with particular blocks and at the end of each sequence, participants were presented with one of the odors from that sequence and asked to indicate the block associated with that odor (note that in this condition, participants did not have to remember the temporal order of presented smells, just their relative locations); And *Semantic-olfactory CBTT*, where participants first heard the list of odorant names, then were presented with a sequence of odors, and then were asked to reproduce the sequence, by naming each odor and touching the correct block (note, that in this condition participants had to recognize the odors and remember the temporal sequence). The performance was measured in terms of sequence length (memory span) before participants committed three errors in a row. Results showed that both athletes and non-athletes performed best in the classic CBTT, and worst in the Semantic-olfactory CBTT. Non-athletes outperformed athletes in all three tests. The authors suggest that poor performance in Semantic-olfactory CBTT could be due to different processing systems involved in semantic versus non-semantic tasks.

Jacobs et al. (2015) asked participants to memorize location in a room based only on their sense of vision, sense of smell, or partially sensory deprived (control condition: wearing blindfold, earplugs and sound-reducing headphones, and nose clips). Specifically, in the olfactory condition, participants were deprived of vision and hearing, and asked to memorize a target location in the room based on the strength and composition of the odors in that particular location. Then, participants were led to another location, sensory covers were removed, and participants were asked to return to the target location. Two jars containing distinct smells were located at midlines of two perpendicular walls creating a 2-dimensional odor gradient in the room, used for navigation in the olfactory task. Other conditions were analogous but used different sensory covers. Results showed that participants were the most accurate in the visual condition (ceiling effects), and least accurate in the control (sensory deprived) condition. Performance in the olfactory condition was better than in the control condition, and better than the average random distance between two random locations in the room. The authors suggest that these results provide a demonstration that humans can use olfaction to map the space and navigate to a memorized location, even with 2-dimensional odor mixtures.

In a study outlining new methods for olfactory-cognitive training, Olofsson et al. (2017) tested participants in two versions of a digitized memory game. In one version, the task was to match pairs of odors, and in the other version the task was to match pairs of visual symbols. Odors and symbols had their specific locations on the screen. Then, participants trained their abilities in the olfactory memory game for eight sessions, and finally, were tested again on both olfactory and visual memory games. Results showed that in both sessions participants performed slightly better in the visual than olfactory game but these comparisons were not tested statistically. Furthermore, the performance improved in both modalities after olfactory training, suggesting that possible benefits from olfactory training might transfer to other perceptual and cognitive domains. However, the authors pointed out that there was no control group in the experiment, so transfer effects may be caused by other reasons, such as task familiarity.

As an expansion to the previous study, Olofsson et al. (2020) tested intramodal and crossmodal transfer effects of training in either olfactory or visual sensory modality. All participants first performed both visual and olfactory memory games (similar to Olofsson et al., 2017, but on a table rather than on a computer screen). Then, participants were randomly divided into two groups, and trained on either the visual or olfactory memory game at home for 40 days. Finally, all participants were again tested on memory games in both modalities. Results showed that performance, measured as a number of trials needed to complete the game, did not differ between the modalities in the pre-training test. Furthermore, participants that trained in the olfactory game showed improvement in both olfactory and memory games during the post-training session (in line with Olofsson et al., 2017), but participants that trained in the visual game only showed improvement in the visual game. The authors suggest that olfactory training might promote cross-modal transfer of cognitive abilities, which is facilitated through the direct and strong connection between olfactory and cognitive processes.

In a study by Ponce et al. (2024), participants were asked to memorize odor-place associations while walking inside a room, and later recall locations of odors either by walking around that room and marking the correct locations using augmented reality (bringing a tablet to a correct location), or by pointing on a 2-dimensional map shown on the screen (top-down view). In the “in-room” recall, participants were allowed three chances to place the odor in its correct location. Performance was measured in terms of a number of correctly placed odors (Total stimuli) in both spatio-contextual environments (in-room and on-screen). Additionally, in the in-room recall, performance was measured as a number of failed attempts in the entire task (Total attempts), time it took for the participants to learn the odor-place association (Learning time), and the time it took for participants to place all odors in their recalled locations (Evaluation time). Performance in the olfactory spatial memory

experiment in this study was compared with performance in visual and tactile spatial memory experiments run by the same research group and with a similar experimental paradigm (Munoz-Montoya et al., 2021). When comparing performance across sensory modalities in the “in-room” recall, results showed that participants needed fewer attempts to correctly place odors in comparison to tactile stimuli, but they needed more time to learn the location of odors than tactile stimuli. Furthermore, participants needed more time to learn and to recall the location of odors, in comparison to the visual stimuli. However, authors point out that the difference in time needed to memorize or recall locations is confounded by the physical and mechanical features of accessing the stimuli (e.g., in the olfactory condition, participants needed to pick up the jar, unscrew the lid, smell, screw the lid, and put the jar back). When comparing performance across sensory modalities in the “on-screen” recall, results showed more correctly placed odors than tactile stimuli, but no statistically significant difference between odors and visual stimuli. Altogether, the authors conclude that these results are in line with previous research showing a similar ability to memorize olfactory, visual, and auditory stimuli (Hamburger & Knauff, 2019), and that olfactory spatial memory is superior to its tactile counterpart.

Participants in five studies by the same research group (Plailly et al., 2019; Rey et al., 2023; Saive et al., 2013, 2014, 2015) were presented with odor-context-place associations, without explicitly being asked to memorize them. Specific design differed between studies, such that n four studies contexts and places within those contexts were presented on a computer screen (Plailly et al., 2019, Rey et al., 2023, Saive et al., 2014, 2015), and in two studies presented in one article, contexts were presented on a computer screen, and places were presented on a table in front of the screen (Saive et al., 2013), Note that in study by Rey, et al. (2023), the authors labeled the on-screen presentation as a “non-immersive virtual reality”. In all studies, during the recall stage, participants were presented with an odor, and if they recognized the odor, they were asked to choose the correct context and then - the correct place for that odor. In four studies, the responses of participants were grouped into four possible combinations: WWW (What-Where-Which)—correct recall of the odor-context-place association, WWhere (What-Where)—correct odor-place association, WWhich (What-Which)—correct odor-context association, What—correct odor recognition (Plailly et al., 2019; Saive et al., 2013, 2014, 2015), and outcome was measured as a number or proportion of responses belonging to each category. In the most recent study, the outcome was measured as a probability to reach each recall step: Recognizing the odor, recalling in which room it was presented, and recalling its location within that room (Rey et al., 2023). In general, the results showed that when faced with an odor-context-place association, if the encoding was successful, participants remembered the entire association rather than single odor-place or odor-context pairs. Furthermore, the recall of the entire

association seemed to happen simultaneously with the recognition of the odor. Such results led the authors to postulate that a singular brain process might underlie both odor recognition and odor-context-place association memory.

Specifically, in the first research article (Saive et al., 2013), the results showed a larger proportion of the WWW and What responses than WWhich and WWhere responses, and proportion of WWhere responses was either very small (study 2) or none (study 1). Therefore, the authors argue that participants either memorized the entire WWW combination or they were unable to recall any association, and that odor-place association (WWhere) was the most difficult to encode.

In the following study (Saive et al., 2014), results showed that proportion of WWW, WWhich, and WWhere responses differed from chance, and the proportion of WWhere responses was significantly lower than any other responses. The authors argue that once we are able to recognize the odor, we are also able to recall the entire odor-context-place association or odor-context association. Again, the odor-place association seemed to be the most difficult to encode. Furthermore, participants' reaction time was measured, first—from the onset of the olfactory stimuli to the odor recognition response, and then—from the recognition response to a response indicating whether the participant remembers the context or not. Results showed that in the second interval, the reaction time was fastest when participants correctly recalled the entire association (WWW), slower for partially recalled association (WWhich), and slowest when participants only recognized the odor (What). This was interpreted by the authors as an indication that a singular memory process underlies both odor-recognition and odor-context-place association, meaning that if the encoding of the odor-context-place association is successful, the recall of that association is simultaneous with the odor recognition. Finally, this study investigated whether pleasantness of the odor affects the memory recall. Results showed better recognition and memory retrieval for pleasant and unpleasant odors, rather than neutral odors. This suggests that it might rather be the emotional arousal, rather than valence, that affects memory performance.

Results of the subsequent study by the same research group (Saive et al., 2015) showed a larger number of WWW and What responses than WWhich and WWhere responses, and the number of WWW responses was the only one higher than chance. Furthermore, after recognizing the odor, participants reported whether they remember the whole episode, or if the odor just feels familiar, and results showed that the number of WWW responses was larger than chance only when participants reported remembering the entire episode. These results were in line with the conclusions from previous studies (Saive et al., 2013, 2014), supporting the notion that odor-recognition and odor-context-place memory originate from

the singular memory process. Notably, the WWhere response only occurred once for one participant, similar to previous studies.

In the next study (Plailly et al., 2019), results showed a larger proportion of WWhich than WWW responses in contrast to previous findings. However, there were almost no proportion of WWhere responses trials, reinforcing the notion from all previous studies that encoding of spatial location is most difficult (Saive et al., 2013, 2014, 2015). Notably, the authors do not focus discussion on these results, because the aim of this study was to test how these visual-olfactory memory episodes are incorporated into participants' dreams, and how that relates to memory performance. Interestingly, odors (unlike visual context) were rarely incorporated into the dreams, and performance in the episodic memory task that included odors, did not differ between participants that reported dreams related to learning and the experiment, and those who did not.

More recently, the research group (Rey et al., 2023), expanded the experimental protocol by comparing participants’ performance across different sensory modalities (olfaction, vision, and hearing). Results showed that participants were most likely to recall the entire object-context-place association for visual objects, then for odors, and least likely for auditory objects. Interestingly, response time for the entire trial (counting from object presentation to place recall) was fastest for trials, in which participants correctly recalled the entire object-context-place association, in line with results from the previous study (Saive et al., 2014). This outcome strengthened the idea that after successful encoding of the odor-context-place association, the recall of the entire association is simultaneous with the odor recognition, and it fuels the authors’ claim that there is a singular memory process that underlies odor recognition and odor-context-place associations.

Raithel et al. (2023) investigated brain activity during an olfactory spatial memory task. Participants were asked to memorize locations of specific odors and use these odors as landmarks, to find a target odor. The results showed that humans can navigate using odors as landmarks, and performance can be improved with training. Performance was not affected by intensity, familiarity, and pleasantness of odors, inhalation duration, or gender. Furthermore, the authors found indication of grid-cells activation in the brain’s higher cognitive areas responsible for spatial memory and navigation, validating the claims of Hamburger and Knauff (2019) and Schwarz and Hamburger (2023) that humans can use odors to build cognitive maps. Finally, similar activity related to spatial encoding was also found in the primary olfactory areas in the brain, suggesting that olfactory primary areas belong to a functional network that relies on olfactory cues during navigation, in line with recent results from animal studies (Poo, et al., 2022).

Schifferstein et al. (2009) compared performance in a spatial memory task between four sensory modalities: vision, hearing, touch, and smell. Different groups of participants

navigated in a room without being explicitly told to memorize locations of the objects they encountered. Then, participants were asked to recall locations of distinct sensory objects in a room. Results showed no statistically significant difference between the sensory conditions in the precise location recollection, however, the authors argue for a slight dominance of vision above other modalities.

Szychowska et al., (2025) investigated effects of retroactive interference between olfaction and hearing in spatial memory task. Participants had to memorize locations of 4 smells and 4 sounds that were conceptually matched (e.g., smell of a coffee and sound of a coffee brewing), in the virtual arena displayed through head mounted display (VR), and then recall them again in the VR at two occasions, after 15-minute delay, and after 1-week delay. Half of the participants started the experiment with encoding locations of smells, and the other half first encoded location of sounds. Results showed overall sensory retroactive interference in the 15-minute delay recall. Specifically, location of objects encoded second was recalled better than those encoded first, regardless of the sensory modality. Results showed no better retention of smell versus sound locations in the 1-week recall, challenging the idea that olfaction does not differentiate between short- and long-term memory. Explorative results showed that in error trials participants tended to place sounds in the location of smells related to the same concept, but not vice-versa, suggesting possible olfactory-specific interference at the conceptual level.

Finally, in two studies, Takahashi (2003) presented participants with different odors in two different rooms. Some participants were told to memorize just the odors, some were told to memorize odor and the room in which the odor was presented. Both groups of participants were later asked to recognize the odor and associate it with the correct room. In the first study, the rooms were similar to each other, in the second study the rooms were made more distinct from each other. Results showed that participants that were only asked to memorize odors performed at a chance level in the odor-place association task, in contrast to participants that were explicitly asked to memorize odor-place pairs. Because the second study resulted in a larger difference between the groups than the first study, the authors argue that increasing distinctiveness of the location improves the performance in the spatial memory task.
