# Supplementary material for: Olfactory spatial memory: a systematic review and meta-analysis
Source: Sci Rep. 2025 Nov 4;15:38469. doi: 10.1038/s41598-025-25503-5 (PMC12586518; doi:10.1038/s41598-025-25503-5)
Supplement: Supplementary file 3 — Supplementary Material 3 [file 41598_2025_25503_MOESM3_ESM.pdf]

Demographic characteristics details

Figure 2 in the manuscript depicts demographic characteristics of the total population in each study (across all experimental groups) and average across studies. For detailed demographic summary per experimental group within each study, see Supplementary Figure 2. On average, studies tested 68 participants (SD = 104, median = 40), 57.5% of participants were female, and average age of participants was 26 years old (SD = 5.81). Most of the studies reported mean and SD or standard error of the mean (SEM) for the age. For those who did not, we estimated values whenever possible.

Seven studies reported age and gender information divided into different sample groups but did not provide a global summary(de Vries, de Vet, et al., 2020; de Vries, Morquecho-Campos, et al., 2020; Gilbert et al., 2008; Invitto et al., 2022; Olofsson et al., 2020; Saive et al., 2013 - study 2; Schifferstein et al., 2009). For these studies, the pooled means and SDs for age shown on Figure 2 were calculated according to the formulas presented in the supplementary material. In two articles, authors reported results of two distinct studies(Saive et al., 2013; Takahashi, 2003), and the descriptive information for each is shown on Figure 2.

In two studies, Takahashi (2003) reported descriptive information for participants before dividing them into two groups: One group that was asked to memorize odor-place associations, and one that was asked to just remember odors, even though both groups were later tested on both odor recognition and odor-place association. Full sample size in each study was 40 participants, later divided randomly into two groups (it is unclear whether groups were of equal size). In both studies only age range was provided. In the first study the author reported that all 40 participants except one were between 19 and 20 years old, and the one participant was 25 years old but did not report the mean and SD. In the second study, all participants were reported to be between 19 and 20 years old.

Olofsson et al. (2017) reported descriptive information for the initial participants sample (shown on Figure 2), but not for the tested sample, after two participants were excluded. In several studies by Hamburger and colleagues (Arena & Hamburger, 2023; Schwarz & Hamburger, 2023a, 2023b) demographic information was reported for the full sample (Figure 2), but participants were later divided into two or three groups that performed the tasks in different sensory conditions. Similarly, Szychowska et al. (Szychowska et al., 2025)(2025) reported demographic information for the entire sample but did not report those values for individual groups (however, because it was study from our research group and all data are available on OSF, we were able to reconstruct that information). Finally, Arena and Hamburger (2023) did not provide information about the gender distribution and reported that two participants dropped out for the second testing session (after one month) but did not provide the demographic information for that session.

References:

Arena, E., & Hamburger, K. (2023). Olfactory and visual vs. multimodal landmark processing in human wayfinding: A virtual reality experiment. *Journal of Cognitive Psychology*, *35*(6–7), 688–709. https://doi.org/10.1080/20445911.2023.2248685

de Vries, R., de Vet, E., de Graaf, K., & Boesveldt, S. (2020). Foraging minds in modern environments: High-calorie and savory-taste biases in human food spatial memory. *Appetite*, *152*. Scopus. https://doi.org/10.1016/j.appet.2020.104718

de Vries, R., Morquecho-Campos, P., de Vet, E., de Rijk, M., Postma, E., de Graaf, K., Engel, B., & Boesveldt, S. (2020). Human spatial memory implicitly prioritizes high-calorie foods. *Scientific Reports*, *10*(1). Scopus. https://doi.org/10.1038/s41598-020-72570-x

Gilbert, P. E., Pirogovsky, E., Ferdon, S., Brushfield, A. M., & Murphy, C. (2008). Differential Effects of Normal Aging on Memory for Odor-Place and Object-Place Associations. *Experimental Aging Research*, *34*(4), 437–452. https://doi.org/10.1080/03610730802271914

Invitto, S., Accogli, G., Leucci, M., Salonna, M., Serio, T., Fancello, F., Ciccarese, V., & Lankford, D. (2022). Spatial Olfactory Memory and Spatial Olfactory Navigation, Assessed with a Variant of Corsi Test, Is Modulated by Gender and Sporty Activity. *Brain Sciences*, *12*(8). Scopus. https://doi.org/10.3390/brainsci12081108

Olofsson, J. K., Ekström, I., Lindström, J., Syrjänen, E., Stigsdotter-Neely, A., Nyberg, L., Jonsson, S., & Larsson, M. (2020). Smell-based memory training: Evidence of Olfactory learning and transfer to the visual domain. *Chemical Senses*, *45*(7), 593–600. Scopus. https://doi.org/10.1093/chemse/bjaa049

Olofsson, J. K., Niedenthal, S., Ehrndal, M., Zakrzewska, M., Wartel, A., & Larsson, M. (2017). Beyond Smell-O-Vision: Possibilities for Smell-Based Digital Media. *Simulation & Gaming*, *48*(4), 455–479. https://doi.org/10.1177/1046878117702184

Saive, A.-L., Ravel, N., Thévenet, M., Royet, J.-P., & Plailly, J. (2013). A novel experimental approach to episodic memory in humans based on the privileged access of odors to memories. *Journal of Neuroscience Methods*, *213*(1), 22–31. https://doi.org/10.1016/j.jneumeth.2012.11.010

Schifferstein, H. N. J., Smeets, M. A., & Postma, A. (2009). Comparing location memory for 4 sensory modalities. *Chemical Senses*, *35*(2), 135–145. Scopus. https://doi.org/10.1093/chemse/bjp090

Schwarz, M., & Hamburger, K. (2023a). Implicit versus explicit processing of visual, olfactory, and multimodal landmark information in human wayfinding. *Frontiers in Psychology*, *14*, 1285034. https://doi.org/10.3389/fpsyg.2023.1285034

Schwarz, M., & Hamburger, K. (2023b). Memory effects of visual and olfactory landmark information in human wayfinding. *Cognitive Processing*. https://doi.org/10.1007/s10339-023-01169-7

Szychowska, M., Ersson, K., & Olofsson, J. K. (2025). Asymmetric cross-sensory interference between spatial memories of sounds and smells revealed in a virtual reality environment. *Journal of Experimental Psychology: Learning, Memory, and Cognition*. https://doi.org/10.1037/xlm0001493

Takahashi, M. (2003). Recognition of odors and identification of sources. *The American Journal of Psychology*, *116*(4), 527–542. https://doi.org/10.2307/1423659
