# Supplementary material for: Olfactory spatial memory: a systematic review and meta-analysis
Source: Sci Rep. 2025 Nov 4;15:38469. doi: 10.1038/s41598-025-25503-5 (PMC12586518; doi:10.1038/s41598-025-25503-5)

Supplement to the manuscript
Olfactory spatial memory: A systematic review and meta-analysis

Supplement Figure 1. Number of peer-reviewed articles published per 5-year bin on olfactory spatial memory.

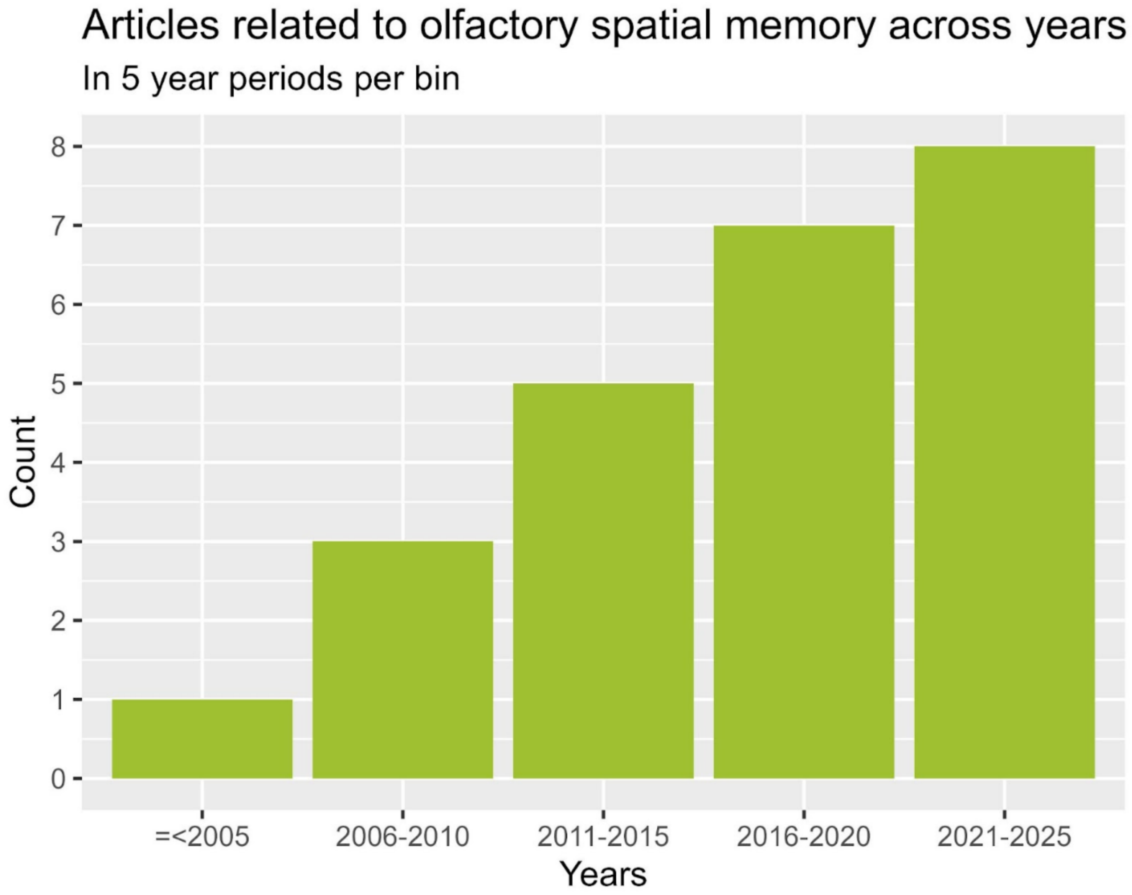

Supplement Table 1. Terms used in the search strategy and in defining inclusion and exclusion criteria, presented in the PICO framework. Note that the "Comparison" column is empty, as limiting the review to comparisons between senses was not the purpose of this systematic review.

**Population**	**Intervention**	**Comparison**	**Outcome**
Adult population	Aroma		Allocentric memory
Healthy adults	Fragrance		Contextual memory
Healthy human subjects	Odor		Cognitive mapping
Non-clinical adults	Olfaction		Environmental memory
Normal adults	Olfactory cue		Location memory
Young adults	Olfactory function		Navigation memory
	Olfactory information		Object location association
	Olfactory perception		Object location memory
	Olfactory sensation		Object-location association
	Olfactory sense		Object-location memory
	Olfactory signal		Object place association
	Olfactory stimulus		Object place memory
	Scent		Object-place association
	Sense of smell		Object-place memory
	Smell		Place memory
			Sensory spatial memory
			Spatial learning
			Spatial memory
			Spatial navigation
			Spatial recall
			Spatial recognition
			Topographical memory
			Wayfinding memory

Supplement Table 2. Search strategies for the three databases

Database	Search strategy
PubMed	(Aroma[Title/Abstract] OR fragance[Title/Abstract] OR odor[Title/Abstract] OR odour[Title/Abstract] OR olfaction[Title/Abstract] OR "olfactory cue"[Title/Abstract] OR "olfactory function"[Title/Abstract] OR "olfactory information"[Title/Abstract] OR "olfactory perception"[Title/Abstract] OR "olfactory sensation"[Title/Abstract] OR "Olfactory sense"[Title/Abstract] OR "olfactory signal"[Title/Abstract] OR "olfactory stimulus"[Title/Abstract] OR scent[Title/Abstract] OR "sense of smell"[Title/Abstract] OR Smell[Title/Abstract]) AND ("allocentric memory"[Title/Abstract] OR "contextual memory"[Title/Abstract] OR " cognitive mapping"[Title/Abstract] OR "environmental memory"[Title/Abstract] OR "location memory"[Title/Abstract] OR "Navigation memory"[Title/Abstract] OR "object location association"[Title/Abstract] OR "object location memory"[Title/Abstract] OR "object*location association"[Title/Abstract] OR "object*location memory"[Title/Abstract] OR "object*place association"[Title/Abstract] OR "object*place memory"[Title/Abstract] OR "Place memory"[Title/Abstract] OR "Sensory spatial memory"[Title/Abstract] OR "Spatial learning"[Title/Abstract] OR "Spatial memory"[Title/Abstract] OR "Spatial navigation"[Title/Abstract] OR "Spatial recall"[Title/Abstract] OR "Spatial recognition"[Title/Abstract] OR "Topographical memory"[Title/Abstract] OR "Wayfinding memory"[Title/Abstract])
WoS	(TS=(Aroma OR fragance OR odor OR odour OR olfaction OR "olfactory cue" OR "olfactory function" OR "olfactory information" OR "olfactory perception" OR "olfactory sensation" OR "Olfactory sense" OR "olfactory signal" OR "olfactory stimulus" OR scent OR "sense of smell" OR Smell)) AND TS=("allocentric memory" OR "contextual memory" OR " cognitive mapping" OR "environmental memory" OR "location memory" OR "Navigation memory" OR "object location association" OR "object location memory" OR "object*location association" OR "object*location memory" OR "object*place association" OR "object*place memory" OR "Place memory" OR "Sensory spatial memory" OR "Spatial learning" OR "Spatial memory" OR "Spatial navigation" OR "Spatial recall" OR "Spatial recognition" OR "Topographical memory" OR "Wayfinding memory") and English (Languages) and Article (Document Types)
Scopus	(TITLE-ABS (Aroma OR fragance OR odor OR odour OR olfaction OR "olfactory cue" OR "olfactory function" OR "olfactory information" OR "olfactory perception" OR "olfactory sensation" OR "Olfactory sense" OR "olfactory signal" OR "olfactory stimulus" OR scent OR "sense of smell" OR Smell)) AND (TITLE-ABS ("allocentric memory" OR "contextual memory" OR " cognitive mapping" OR "environmental memory" OR "location memory" OR "Navigation memory" OR "object location association" OR "object location memory" OR "object*location association" OR "object*location memory" OR "object*place association" OR "object*place memory" OR "Place memory" OR "Sensory spatial memory" OR "Spatial learning" OR "Spatial memory" OR "Spatial navigation" OR "Spatial recall" OR "Spatial recognition" OR "Topographical memory" OR "Wayfinding memory")) AND (LIMIT-TO (LANGUAGE , "English")) AND (LIMIT-TO (DOCTYPE , "ar"))

Supplement Table 3. List of studies included in the systematic review with indication whether they were found in the original search or by reference tracking.

Study	Identification through…
1. Arena & Hamburger, 2023	Reference tracking
2. Brünner, et al., 2015	Original search
3. Brünner, et al., 2016	Original search
4. de Vries, de Vet, et al., 2020	Original search
5. de Vries, Morquecho-Campos, et al., 2020	Original search
6. Gilbert et al., 2008	Original search
7. Goodrich-Hunsaker et al., 2009	Original search
8. Hamburger & Knauff, 2019	Original search
9. Invitto et al., 2022	Original search
10. Jacobs et al., 2015	Original search
11. Olofsson et al., 2017	Reference tracking
12. Olofsson et al., 2020	Original search
13. Plailly et al., 2019	Original search
14. Ponce et al., 2024	Original search
15. Raithel et al., 2023	Original search
16. Rey et al., 2023	Reference tracking
17. Saive et al., 2013	Original search
18. Saive et al., 2014	Reference tracking
19. Saive et al., 2015	Reference tracking
20. Schifferstein et al., 2009	Original search
21. Schwarz & Hamburger, 2023a	Reference tracking
22. Schwarz & Hamburger, 2023b	Reference tracking
23. Szychowska et al., 2025	Authors own empirical study published during peer-review process of this systematic review
24. Takahashi, 2003	Reference tracking

References:

Arena, E., & Hamburger, K. (2023). Olfactory and visual vs. multimodal landmark processing in human wayfinding: A virtual reality experiment. *Journal of Cognitive Psychology*, *35*(6–7), 688–709. https://doi.org/10.1080/20445911.2023.2248685

Brünner, Y. F., Kofoet, A., Benedict, C., & Freiherr, J. (2015). Central insulin administration improves odor cued reactivation of spatial memory in young men. *Journal of Clinical Endocrinology and Metabolism*, *100*(1), 212–219. Scopus. https://doi.org/10.1210/jc.2014-3018

Brünner, Y. F., Rodriguez-Raecke, R., Mutic, S., Benedict, C., & Freiherr, J. (2016). Neural correlates of olfactory and visual memory performance in 3D-simulated mazes after intranasal insulin application. *Neurobiology of Learning and Memory*, *134*, 256–263. https://doi.org/10.1016/j.nlm.2016.07.027

de Vries, R., de Vet, E., de Graaf, K., & Boesveldt, S. (2020). Foraging minds in modern environments: High-calorie and savory-taste biases in human food spatial memory. *Appetite*, *152*. Scopus. https://doi.org/10.1016/j.appet.2020.104718

de Vries, R., Morquecho-Campos, P., de Vet, E., de Rijk, M., Postma, E., de Graaf, K., Engel, B., & Boesveldt, S. (2020). Human spatial memory implicitly prioritizes high-calorie foods. *Scientific Reports*, *10*(1). Scopus. https://doi.org/10.1038/s41598-020-72570-x

Gilbert, P. E., Pirogovsky, E., Ferdon, S., Brushfield, A. M., & Murphy, C. (2008). Differential Effects of Normal Aging on Memory for Odor-Place and Object-Place Associations. *Experimental Aging Research*, *34*(4), 437–452. https://doi.org/10.1080/03610730802271914

Goodrich-Hunsaker, N. J., Gilbert, P. E., & Hopkins, R. O. (2009). The role of the human hippocampus in odor-place associative memory. *Chemical Senses*, *34*(6), 513–521. Scopus. https://doi.org/10.1093/chemse/bjp026

Hamburger, K., & Knauff, M. (2019). Odors Can Serve as Landmarks in Human Wayfinding. *Cognitive Science*, *43*(11), e12798. https://doi.org/10.1111/cogs.12798

Invitto, S., Accogli, G., Leucci, M., Salonna, M., Serio, T., Fancello, F., Ciccarese, V., & Lankford, D. (2022). Spatial Olfactory Memory and Spatial Olfactory Navigation, Assessed with a Variant of Corsi Test, Is Modulated by Gender and Sporty Activity. *Brain Sciences*, *12*(8). Scopus. https://doi.org/10.3390/brainsci12081108

Jacobs, L. F., Arter, J., Cook, A., & Sulloway, F. J. (2015). Olfactory orientation and navigation in humans. *PLoS ONE*, *10*(6). Scopus. https://doi.org/10.1371/journal.pone.0129387

Olofsson, J. K., Ekström, I., Lindström, J., Syrjänen, E., Stigsdotter-Neely, A., Nyberg, L., Jonsson, S., & Larsson, M. (2020). Smell-based memory training: Evidence of Olfactory learning and transfer to the visual domain. *Chemical Senses*, *45*(7), 593–600. Scopus. https://doi.org/10.1093/chemse/bjaa049

Olofsson, J. K., Niedenthal, S., Ehrndal, M., Zakrzewska, M., Wartel, A., & Larsson, M. (2017). Beyond Smell-O-Vision: Possibilities for Smell-Based Digital Media. *Simulation & Gaming*, *48*(4), 455–479. https://doi.org/10.1177/1046878117702184

Plailly, J., Villalba, M., Vallat, R., Nicolas, A., & Ruby, P. (2019). Incorporation of fragmented visuo-olfactory episodic memory into dreams and its association with memory performance. *Scientific Reports*, *9*(1). Scopus. https://doi.org/10.1038/s41598-019-51497-y

Ponce, D., Torres, C., Mendez-Lopez, M., Molla, R., & Juan, M.-C. (2024). Augmented Reality to Assess Short-Term Spatial Memory: A Comparative Study of Olfactory, Visual, and Tactile Stimuli. *IEEE Access*, *12*, 47041–47056. Scopus. https://doi.org/10.1109/ACCESS.2024.3382251

Raithel, C. U., Miller, A. J., Epstein, R. A., Kahnt, T., & Gottfried, J. A. (2023). Recruitment of grid-like responses in human entorhinal and piriform cortices by odor landmark-based navigation. *Current Biology*, S0960982223008734. https://doi.org/10.1016/j.cub.2023.06.087

Rey, L., Désoche, C., Saive, A.-L., Thévenet, M., Garcia, S., Tillmann, B., & Plailly, J. (2023). Episodic memory and recognition are influenced by cues' sensory modality: Comparing odours, music and faces using virtual reality. *Memory*, *31*(9), 1113–1133. https://doi.org/10.1080/09658211.2023.2208793

Saive, A.-L., Ravel, N., Thévenet, M., Royet, J.-P., & Plailly, J. (2013). A novel experimental approach to episodic memory in humans based on the privileged access of odors to memories. *Journal of Neuroscience Methods*, *213*(1), 22–31. https://doi.org/10.1016/j.jneumeth.2012.11.010

Saive, A.-L., Royet, J.-P., Garcia, S., Thévenet, M., & Plailly, J. (2015). "What-Where-Which" Episodic Retrieval Requires Conscious Recollection and Is Promoted by Semantic Knowledge. *PLOS ONE*, *10*(12), e0143767. https://doi.org/10.1371/journal.pone.0143767

Saive, A.-L., Royet, J.-P., Ravel, N., Thévenet, M., Garcia, S., & Plailly, J. (2014). A unique memory process modulated by emotion underpins successful odor recognition and episodic retrieval in humans. *Frontiers in Behavioral Neuroscience*, *8*. https://www.frontiersin.org/articles/10.3389/fnbeh.2014.00203

Schifferstein, H. N. J., Smeets, M. A., & Postma, A. (2009). Comparing location memory for 4 sensory modalities. *Chemical Senses*, *35*(2), 135–145. Scopus. https://doi.org/10.1093/chemse/bjp090

Schwarz, M., & Hamburger, K. (2023a). Implicit versus explicit processing of visual, olfactory, and multimodal landmark information in human wayfinding. *Frontiers in Psychology*, *14*, 1285034. https://doi.org/10.3389/fpsyg.2023.1285034

Schwarz, M., & Hamburger, K. (2023b). Memory effects of visual and olfactory landmark information in human wayfinding. *Cognitive Processing*. https://doi.org/10.1007/s10339-023-01169-7

Szychowska, M., Ersson, K., & Olofsson, J. K. (2025). Asymmetric cross-sensory interference between spatial memories of sounds and smells revealed in a virtual reality environment. *Journal of Experimental Psychology: Learning, Memory, and Cognition*. https://doi.org/10.1037/xlm0001493

Takahashi, M. (2003). Recognition of Odors and Identification of Sources. *The American Journal of Psychology*, *116*(4), 527–542. https://doi.org/10.2307/1423659

Supplement Table 4. List of studies excluded during full-text screening, together with the exclusion reasons.

	Exclusion reason		
**Study**	**Study design**	**Population**	**Outcome**
Barnett, et al., 1999			No olfactory spatial memory task
Beer, et al., 2013		Animal population	
Belnoue, et al., 2011		Animal population	No olfactory spatial memory task
Dahmani, et al., 2020			No olfactory spatial memory task
Dahmani, et al., 2018			No olfactory spatial memory task
Doty, et al., 2015			No olfactory spatial memory task
Fischler-Ruiz, et al., 2021		Animal population	
Kinsley, et al., 2012	Review		
Masmudi-Martin, et al., 2019		Animal population	
Merhav, et al., 2019			No olfactory spatial memory task
Merhav & Wolbers, 2019			No olfactory spatial memory task
Muller, et al., 2021			No olfactory spatial memory task
Ozturk, et al., 2020	Survey		No olfactory spatial memory task
Pacharra, et al., 2016			No olfactory spatial memory task
Pool, et al., 2014			No olfactory spatial memory task
Schroder, et al., 2015			No olfactory spatial memory task
Schwabe, et al., 2009			No olfactory spatial memory task
Shanahan, et al., 2018			No olfactory spatial memory task
Skosnik, et al., 2001			No olfactory spatial memory task
Sun, et al., 2016		Animal population	No olfactory spatial memory task
Turetsky, et al., 2003			No olfactory spatial memory task
Zhang, et al., 2015		Animal population	No olfactory spatial memory task

References:

Barnett, R., Maruff, P., Purcell, R., Wainwright, K., Kyrios, M., Brewer, W., & Pantelis, C. (1999). Impairment of olfactory identification in obsessive-compulsive disorder. *Psychological Medicine*, *29*(5), 1227–1233. Scopus. https://doi.org/10.1017/S0033291799008818

Beer, Z., Chwiesko, C., Kitsukawa, T., & Sauvage, M. M. (2013). Spatial and stimulus-type tuning in the LEC, MEC, POR, PrC, CA1, and CA3 during spontaneous item recognition memory. *Hippocampus*, *23*(12), 1425–1438. Scopus. https://doi.org/10.1002/hipo.22195

Belnoue, L., Grosjean, N., Abrous, D., & Koehl, M. (2011). A Critical Time Window for the Recruitment of Bulbar Newborn Neurons by Olfactory Discrimination Learning. *JOURNAL OF NEUROSCIENCE*, *31*(3), 1010–1016. https://doi.org/10.1523/JNEUROSCI.3941-10.2011

Dahmani, L., Courcot, B., Near, J., Patel, R., Amaral, R. S. C., Chakravarty, M. M., & Bohbot, V. D. (2020). Fimbria-Fornix Volume Is Associated With Spatial Memory and Olfactory Identification in Humans. *Frontiers in Systems Neuroscience*, *13*. Scopus. https://doi.org/10.3389/fnsys.2019.00087

Dahmani, L., Patel, R. M., Yang, Y., Chakravarty, M. M., Fellows, L. K., & Bohbot, V. D. (2018). An intrinsic association between olfactory identification and spatial memory in humans. *Nature Communications*, *9*(1). Scopus. https://doi.org/10.1038/s41467-018-06569-4

Doty, R. L., Tourbier, I., Ng, V., Neff, J., Armstrong, D., Battistini, M., Sammel, M. D., Gettes, D., Evans, D. L., Mirza, N., Moberg, P. J., Connolly, T., & Sondheimer, S. J. (2015). Influences of hormone replacement therapy on olfactory and cognitive function in postmenopausal women. *Neurobiology of Aging*, *36*(6), 2053–2059. Scopus. https://doi.org/10.1016/j.neurobiolaging.2015.02.028

Fischler-Ruiz, W., Clark, D. G., Joshi, N. R., Devi-Chou, V., Kitch, L., Schnitzer, M., Abbott, L. F., & Axel, R. (2021). Olfactory landmarks and path integration converge to form a cognitive spatial map. *Neuron*, *109*(24), 4036-4049.e5. Scopus. https://doi.org/10.1016/j.neuron.2021.09.055

Kinsley, C. H., Meyer, E., & Rafferty, K. A. (2012). Sex steroid hormone determination of the maternal brain: Effects beyond reproduction. *Mini-Reviews in Medicinal Chemistry*, *12*(11), 1063–1070. Scopus. https://doi.org/10.2174/138955712802762248

Masmudi-Martin, M., Navarro-Lobato, I., López-Aranda, M. F., Delgado, G., Martín-Montañez, E., Quiros-Ortega, M. E., Carretero-Rey, M., Narváez, L., Garcia-Garrido, M. F., Posadas, S., López-Téllez, J. F., Blanco, E., Jiménez-Recuerda, I., Granados-Durán, P., Paez-Rueda, J., López, J. C., & Khan, Z. U. (2019). RGS14414

treatment induces memory enhancement and rescues episodic memory deficits. *FASEB Journal*, *33*(11), 11804–11820. Scopus. https://doi.org/10.1096/fj.201900429RR

Merhav, M., Riemer, M., & Wolbers, T. (2019). Spatial updating deficits in human aging are associated with traces of former memory representations. *NEUROBIOLOGY OF AGING*, *76*, 53–61. https://doi.org/10.1016/j.neurobiolaging.2018.12.010

Merhav, M., & Wolbers, T. (2019). Aging and spatial cues influence the updating of navigational memories. *SCIENTIFIC REPORTS*, *9*. https://doi.org/10.1038/s41598-019-47971-2

Muller, N., Kohn, N., van Buuren, M., Klijn, N., Emmen, H., Berkers, R., Dresler, M., Janzen, G., & Fernandez, G. (2021). Differences in executive abilities rather than associative processes contribute to memory development. *HUMAN BRAIN MAPPING*, *42*(18), 6000–6013. https://doi.org/10.1002/hbm.25665

Ozturk, A., & Ozturk, S. (2020). The Brand and Sensation Relation as a Spatial Tracking in Shopping Malls. *ICONARP INTERNATIONAL JOURNAL OF ARCHITECTURE AND PLANNING*, *8*(2), 821–844. https://doi.org/10.15320/ICONARP.2020.138

Pacharra, M., Schäper, M., Kleinbeck, S., Blaszkewicz, M., Golka, K., & van Thriel, C. (2016). Neurobehavioral effects of exposure to propionic acid revisited-Does psychosocial stress interfere with distractive effects in volunteers? *NeuroToxicology*, *55*, 102–111. Scopus. https://doi.org/10.1016/j.neuro.2016.05.019

Pool, E., Brosch, T., Delplanque, S., & Sander, D. (2014). Where is the chocolate? Rapid spatial orienting toward stimuli associated with primary rewards. *Cognition*, *130*(3), 348–359. Scopus. https://doi.org/10.1016/j.cognition.2013.12.002

Schroder, T., Haak, K., Jimenez, N., Beckmann, C., & Doeller, C. (2015). Functional topography of the human entorhinal cortex. *ELIFE*, *4*. https://doi.org/10.7554/eLife.06738

Schwabe, L., Bohringer, A., & Wolf, O. (2009). Stress disrupts context-dependent memory. *LEARNING & MEMORY*, *16*(2), 110–113. https://doi.org/10.1101/lm.1257509

Shanahan, L. K., Gjorgieva, E., Paller, K. A., Kahnt, T., & Gottfried, J. A. (2018). Odor-evoked category reactivation in human ventromedial prefrontal cortex during sleep promotes memory consolidation. *eLife*, *7*. Scopus. https://doi.org/10.7554/eLife.39681

Skosnik, P. D., Spatz-Glenn, L., & Park, S. (2001). Cannabis use is associated with schizotypy and attentional disinhibition. *Schizophrenia Research*, *48*(1), 83–92. Scopus. https://doi.org/10.1016/S0920-9964(00)00132-8

Sun, T., Li, T., Davies, H., Li, W., Yang, J., Li, S., & Ling, S. (2016). Altered morphologies and functions of the olfactory bulb and hippocampus induced by miR-30c. *Frontiers in Neuroscience*, *10*(MAY). Scopus. https://doi.org/10.3389/fnins.2016.00207

Turetsky, B. I., Moberg, P. J., Roalf, D. R., Arnold, S. E., & Gur, R. E. (2003). Decrements in volume of anterior ventromedial temporal lobe and olfactory dysfunction in schizophrenia. *Archives of General Psychiatry*, *60*(12), 1193–1200. https://doi.org/10.1001/archpsyc.60.12.1193

Zhang, S., & Manahan-Vaughan, D. (2015). Spatial olfactory learning contributes to place field formation in the hippocampus. *Cerebral Cortex*, *25*(2), 423–432. Scopus. https://doi.org/10.1093/cercor/bht239

Formulas for pooled mean and SD:

$$M = \frac{n_a m_a + n_b m_b}{n_a + n_b},$$

$$SD_{pooled} = \sqrt{\frac{(n_a - 1)SD_a^2 + (n_b - 1)SD_b^2}{n_a + n_b - 2}},$$

where:

n_a = size of the first sample,
m_a = mean score for the first sample,
SD_a = standard deviation score for the first sample,
n_b = size of the second sample,
m_b = mean score for the second sample
SD_b = standard deviation score for the second sample.

Supplement Figure 2. Summary of demographic information for individual experimental groups in all studies and across the studies. First column (left) shows number of participants in individual groups, and median (open circle), mean and SD across all groups on the bottom. Second column (middle) shows color-coded gender distribution in individual groups, and averaged across all groups. Third column (right) shows mean, SD (solid line), and range (dashed line) of participants age in individual groups, and a mean and SD across all studies on the groups.

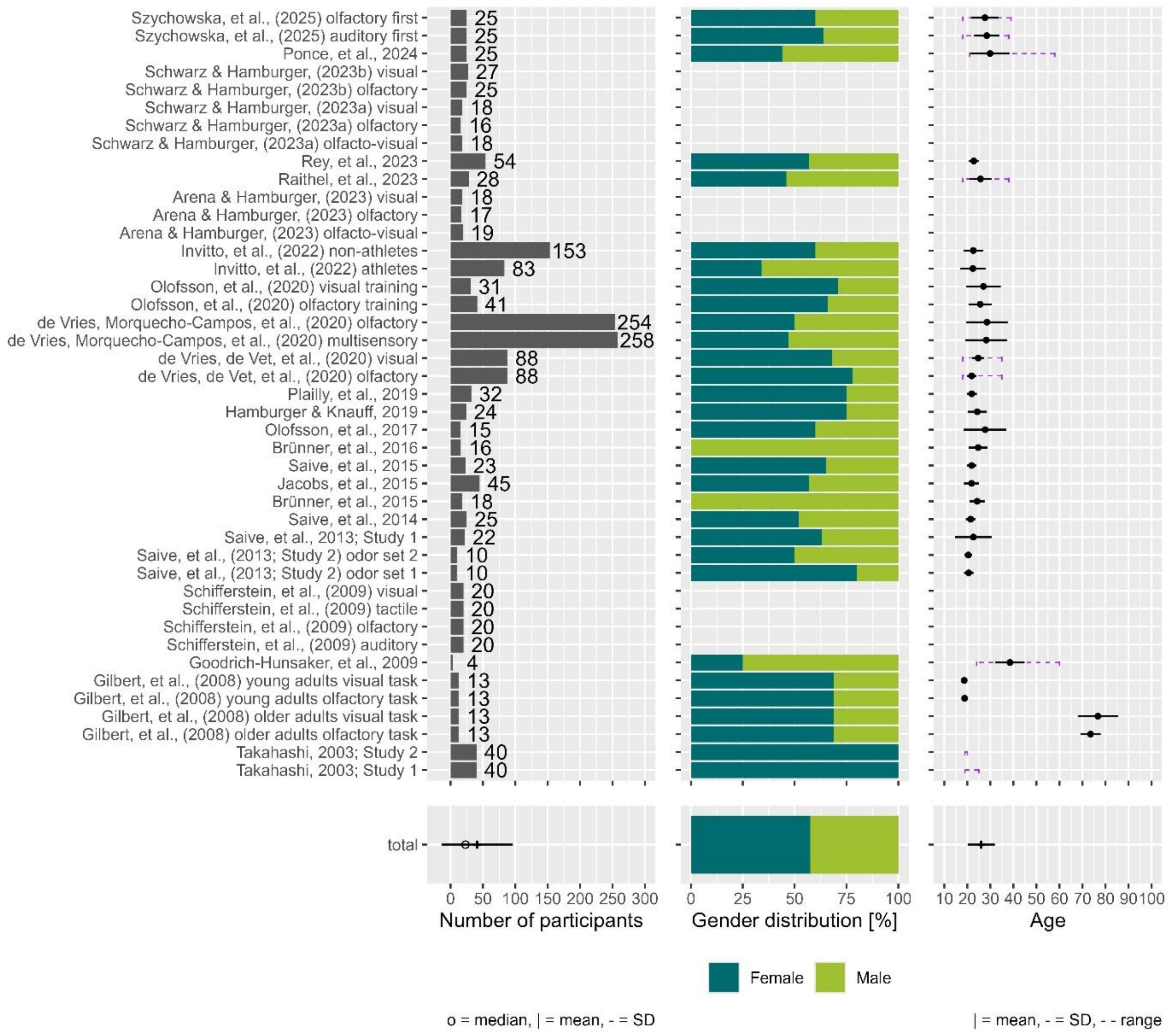

Supplement Table 5. Assessment of risk of bias in individual studies, including a summary score (color coded with **blue** indicating high quality, **orange** – medium quality, and **red** – low quality).

**Study**	**Were the criteria for inclusion in the sample clearly defined?**	**Were the study subjects and the setting described in detail?**	**Were confounding factors identified?**	**Were strategies to deal with confounding factors stated?**	**Were the outcomes measured in a valid and reliable way?**	**Was appropriate statistical analysis used?**	**SUM**
Arena & Hamburger, 2023	1	0	1	1	1	1	5
Brünner et al., 2015	1	1	1	1	1	1	6
Brünner et al., 2016	1	1	1	1	1	1	6
de Vries, de Vet, et al., 2020	1	1	1	1	1	1	6
de Vries, Morquecho-Campos, et al., 2020	0	1	1	1	1	1	5
Gilbert et al., 2008	1	1	1	1	0	1	5
Goodrich-Hunsaker et al., 2009	1	1	1	1	0	1	5
Hamburger & Knauff, 2019	1	1	1	1	1	1	6
Invitto et al., 2022	1	1	0	0	0	1	3
Jacobs et al., 2015	1	1	1	1	1	1	6
Olofsson et al., 2017	0	0	1	1	1	1	4
Olofsson et al., 2022	1	1	1	1	1	1	6
Plailly et al., 2019	1	1	1	1	1	1	6
Ponce et al., 2024	0	1	1	1	1	1	5
Raithel et al., 2023	1	1	1	1	1	1	6
Rey et al., 2023	1	1	1	1	1	1	6
Saive et al., 2013	1	0	1	1	1	1	5
Saive et al., 2014	1	1	1	1	1	1	6
Saive et al., 2015	1	1	1	1	1	1	6
Schifferstein et al., 2009	0	0	1	1	1	1	4
Schwarz & Hamburger, 2023a	1	0	1	1	1	1	5
Schwarz & Hamburger, 2023b	1	0	1	1	1	1	5
Szychowska et al., 2025	1	0	1	1	1	1	5
Takahashi, 2003	1	0	1	1	1	1	5

References:

Arena, E., & Hamburger, K. (2023). Olfactory and visual vs. multimodal landmark processing in human wayfinding: A virtual reality experiment. *Journal of Cognitive Psychology*, *35*(6–7), 688–709. https://doi.org/10.1080/20445911.2023.2248685

Brünner, Y. F., Kofoet, A., Benedict, C., & Freiherr, J. (2015). Central insulin administration improves odor cued reactivation of spatial memory in young men. *Journal of Clinical Endocrinology and Metabolism*, *100*(1), 212–219. Scopus. https://doi.org/10.1210/jc.2014-3018

Brünner, Y. F., Rodriguez-Raecke, R., Mutic, S., Benedict, C., & Freiherr, J. (2016). Neural correlates of olfactory and visual memory performance in 3D-simulated mazes after intranasal insulin application. *Neurobiology of Learning and Memory*, *134*, 256–263. https://doi.org/10.1016/j.nlm.2016.07.027

de Vries, R., de Vet, E., de Graaf, K., & Boesveldt, S. (2020). Foraging minds in modern environments: High-calorie and savory-taste biases in human food spatial memory. *Appetite*, *152*. Scopus. https://doi.org/10.1016/j.appet.2020.104718

de Vries, R., Morquecho-Campos, P., de Vet, E., de Rijk, M., Postma, E., de Graaf, K., Engel, B., & Boesveldt, S. (2020). Human spatial memory implicitly prioritizes high-calorie foods. *Scientific Reports*, *10*(1). Scopus. https://doi.org/10.1038/s41598-020-72570-x

Gilbert, P. E., Pirogovsky, E., Ferdon, S., Brushfield, A. M., & Murphy, C. (2008). Differential Effects of Normal Aging on Memory for Odor-Place and Object-Place Associations. *Experimental Aging Research*, *34*(4), 437–452. https://doi.org/10.1080/03610730802271914

Goodrich-Hunsaker, N. J., Gilbert, P. E., & Hopkins, R. O. (2009). The role of the human hippocampus in odor-place associative memory. *Chemical Senses*, *34*(6), 513–521. Scopus. https://doi.org/10.1093/chemse/bjp026

Hamburger, K., & Knauff, M. (2019). Odors Can Serve as Landmarks in Human Wayfinding. *Cognitive Science*, *43*(11), e12798. https://doi.org/10.1111/cogs.12798

Invitto, S., Accogli, G., Leucci, M., Salonna, M., Serio, T., Fancello, F., Ciccarese, V., & Lankford, D. (2022). Spatial Olfactory Memory and Spatial Olfactory Navigation, Assessed with a Variant of Corsi Test, Is Modulated by Gender and Sporty Activity. *Brain Sciences*, *12*(8). Scopus. https://doi.org/10.3390/brainsci12081108

Jacobs, L. F., Arter, J., Cook, A., & Sulloway, F. J. (2015). Olfactory orientation and navigation in humans. *PLoS ONE*, *10*(6). Scopus. https://doi.org/10.1371/journal.pone.0129387

Olofsson, J. K., Ekström, I., Lindström, J., Syrjänen, E., Stigsdotter-Neely, A., Nyberg, L., Jonsson, S., & Larsson, M. (2020). Smell-based memory training: Evidence of Olfactory learning and transfer to the visual domain. *Chemical Senses*, *45*(7), 593–600. Scopus. https://doi.org/10.1093/chemse/bjaa049

Olofsson, J. K., Niedenthal, S., Ehrndal, M., Zakrzewska, M., Wartel, A., & Larsson, M. (2017). Beyond Smell-O-Vision: Possibilities for Smell-Based Digital Media. *Simulation & Gaming*, *48*(4), 455–479. https://doi.org/10.1177/1046878117702184

Plailly, J., Villalba, M., Vallat, R., Nicolas, A., & Ruby, P. (2019). Incorporation of fragmented visuo-olfactory episodic memory into dreams and its association with memory performance. *Scientific Reports*, *9*(1). Scopus. https://doi.org/10.1038/s41598-019-51497-y

Ponce, D., Torres, C., Mendez-Lopez, M., Molla, R., & Juan, M.-C. (2024). Augmented Reality to Assess Short-Term Spatial Memory: A Comparative Study of Olfactory, Visual, and Tactile Stimuli. *IEEE Access*, *12*, 47041–47056. Scopus. https://doi.org/10.1109/ACCESS.2024.3382251

Raithel, C. U., Miller, A. J., Epstein, R. A., Kahnt, T., & Gottfried, J. A. (2023). Recruitment of grid-like responses in human entorhinal and piriform cortices by odor landmark-based navigation. *Current Biology*, S0960982223008734. https://doi.org/10.1016/j.cub.2023.06.087

Rey, L., Désoche, C., Saive, A.-L., Thévenet, M., Garcia, S., Tillmann, B., & Plailly, J. (2023). Episodic memory and recognition are influenced by cues' sensory modality: Comparing odours, music and faces using virtual reality. *Memory*, *31*(9), 1113–1133. https://doi.org/10.1080/09658211.2023.2208793

Saive, A.-L., Ravel, N., Thévenet, M., Royet, J.-P., & Plailly, J. (2013). A novel experimental approach to episodic memory in humans based on the privileged access of odors to memories. *Journal of Neuroscience Methods*, *213*(1), 22–31. https://doi.org/10.1016/j.jneumeth.2012.11.010

Saive, A.-L., Royet, J.-P., Garcia, S., Thévenet, M., & Plailly, J. (2015). "What-Where-Which" Episodic Retrieval Requires Conscious Recollection and Is Promoted by Semantic Knowledge. *PLOS ONE*, *10*(12), e0143767. https://doi.org/10.1371/journal.pone.0143767

Saive, A.-L., Royet, J.-P., Ravel, N., Thévenet, M., Garcia, S., & Plailly, J. (2014). A unique memory process modulated by emotion underpins successful odor recognition and episodic retrieval in humans. *Frontiers in Behavioral Neuroscience*, *8*. https://www.frontiersin.org/articles/10.3389/fnbeh.2014.00203

Schifferstein, H. N. J., Smeets, M. A., & Postma, A. (2009). Comparing location memory for 4 sensory modalities. *Chemical Senses*, *35*(2), 135–145. Scopus. https://doi.org/10.1093/chemse/bjp090

Schwarz, M., & Hamburger, K. (2023a). Implicit versus explicit processing of visual, olfactory, and multimodal landmark information in human wayfinding. *Frontiers in Psychology*, *14*, 1285034. https://doi.org/10.3389/fpsyg.2023.1285034

Schwarz, M., & Hamburger, K. (2023b). Memory effects of visual and olfactory landmark information in human wayfinding. *Cognitive Processing*. https://doi.org/10.1007/s10339-023-01169-7

Szychowska, M., Ersson, K., & Olofsson, J. K. (2025). Asymmetric cross-sensory interference between spatial memories of sounds and smells revealed in a virtual reality environment. *Journal of Experimental Psychology: Learning, Memory, and Cognition*. https://doi.org/10.1037/xlm0001493

Takahashi, M. (2003). Recognition of Odors and Identification of Sources. *The American Journal of Psychology*, *116*(4), 527–542. https://doi.org/10.2307/1423659

Supplement Figure 3. Summary of the methods in the studies from the meta-analysis.

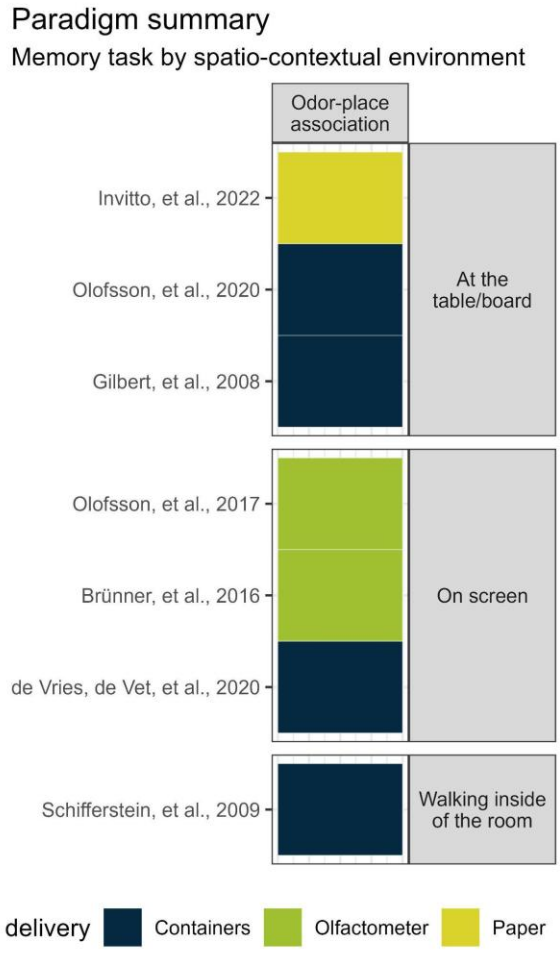

Supplement Table 6. Summary of stimuli types, identifiability, and matching for studies that compared olfactory and visual spatial memory.

Study	Odors	Identifiability of odors	Objects	Identifiability of objects	Stimuli match between sensory modalities
Brünner, et al., 2016	Rose, lavender, flower, lilac, violet, geranium, jasmine, orange, peach, lime, pineapple, melon, strawberry, plum	Unclear, but performance in odor identification task after the memory task was similar to performance of icon identification	Fruity and flowery icons	Unclear, but performance in odor identification task after the memory task was similar to performance of icon identification	Somewhat (all fruits and flowers)
de Vries, de Vet, et al., 2020	Chocolate, caramel, vanilla, apple bake, butter popcorn, roast beef, roasted peanuts, bacon, melon, pineapple, pear, blackcurrant, asparagus, cucumber, tomato, mushroom	Unclear, not reported	Food photographs	Y	Somewhat (all food items)
Gilbert, et al., 2008	Coffee, peppermint, mustard, perfume, banana, cinnamon, garlic, chocolate, soap, vanilla, baby powder, vick vapor rub	Unclear, not reported	Hair brush, plastic frog, stuffed teddy bear, paintbrush, toy helicopter, deck of cards, baseball, calculator, screwdriver, toy squirt gun, 9-volt battery, pad lock	Y	N
Invitto, et al., 2022	Solvent, mint, cinnamon, eucalyptus, cloves, geranium, grass, banana, rose	Unclear, participants asked to label odors after OCBTT, in SOCBTT after getting a list of labels	Not applicable, no visual stimuli, just locations of blocks	Not applicable	Not applicable
Olofsson, et al., 2017	Not reported	Unclear, not reported	Symbols	Unclear	Unclear but most likely N
Olofsson, et al., 2020	Almond, wild strawberry, liquorice, cardamon, vanilla, milk and green tea, rose, peppermint, eucalyptus, lemon, lavender, mango and passion fruit, coconut, rhubarb, smoke, cinnamon, coffee, chocolate, ginger, mulberry, lime, camomile, orange, jasmine	Unclear, not reported	Symbols coming from various languages	N	N
Schifferstein, et al., 2009	Methyl nonyl ketone, petiole 10%, jasmopyrane forte, elinthaal 1%, styrallyl	N; odors chosen for their low identifiability	Shapes	N; shapes chosen for their low identifiability	N (not possible to match

	acetate, cyclamal 10%, cytronellyl acetate, ionone alpha, gyrane, florocyclene ; alcohol C8, ethyl hexanoate, methyl anthranilate 10%, phenyl ethyl methyl ether 10%, phenyl acetaldehyde dimethyl acetal 10%, para cresyl methyl ether, nopylacetate, alicate, anther, pelargene		unidentifiable odors and shapes)

References:

Brünner, Y. F., Rodriguez-Raecke, R., Mutic, S., Benedict, C., & Freiherr, J. (2016). Neural correlates of olfactory and visual memory performance in 3D-simulated mazes after intranasal insulin application. *Neurobiology of Learning and Memory*, *134*, 256–263. https://doi.org/10.1016/j.nlm.2016.07.027

de Vries, R., de Vet, E., de Graaf, K., & Boesveldt, S. (2020). Foraging minds in modern environments: High-calorie and savory-taste biases in human food spatial memory. *Appetite*, *152*. Scopus. https://doi.org/10.1016/j.appet.2020.104718

Gilbert, P. E., Pirogovsky, E., Ferdon, S., Brushfield, A. M., & Murphy, C. (2008). Differential Effects of Normal Aging on Memory for Odor-Place and Object-Place Associations. *Experimental Aging Research*, *34*(4), 437–452. https://doi.org/10.1080/03610730802271914

Invitto, S., Accogli, G., Leucci, M., Salonna, M., Serio, T., Fancello, F., Ciccarese, V., & Lankford, D. (2022). Spatial Olfactory Memory and Spatial Olfactory Navigation, Assessed with a Variant of Corsi Test, Is Modulated by Gender and Sporty Activity. *Brain Sciences*, *12*(8). Scopus. https://doi.org/10.3390/brainsci12081108

Olofsson, J. K., Ekström, I., Lindström, J., Syrjänen, E., Stigsdotter-Neely, A., Nyberg, L., Jonsson, S., & Larsson, M. (2020). Smell-based memory training: Evidence of Olfactory learning and transfer to the visual domain. *Chemical Senses*, *45*(7), 593–600. Scopus. https://doi.org/10.1093/chemse/bjaa049

Olofsson, J. K., Niedenthal, S., Ehrndal, M., Zakrzewska, M., Wartel, A., & Larsson, M. (2017). Beyond Smell-O-Vision: Possibilities for Smell-Based Digital Media. *Simulation & Gaming*, *48*(4), 455–479. https://doi.org/10.1177/1046878117702184

Schifferstein, H. N. J., Smeets, M. A., & Postma, A. (2009). Comparing location memory for 4 sensory modalities. *Chemical Senses*, *35*(2), 135–145. Scopus. https://doi.org/10.1093/chemse/bjp090

Supplement figure 4. Summary of the odor identifiability as reported in the studies.

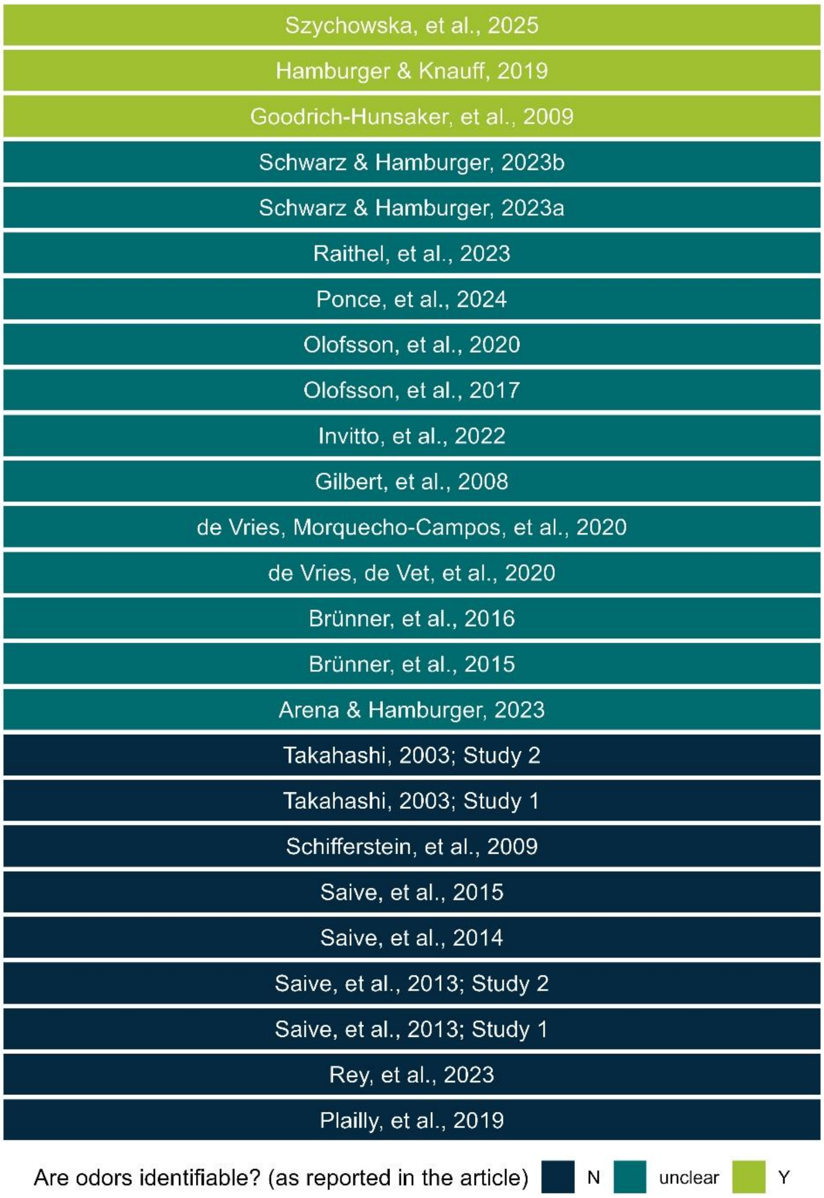

Supplement figure 5. List of the odors reported as difficult to identify (per study and count).

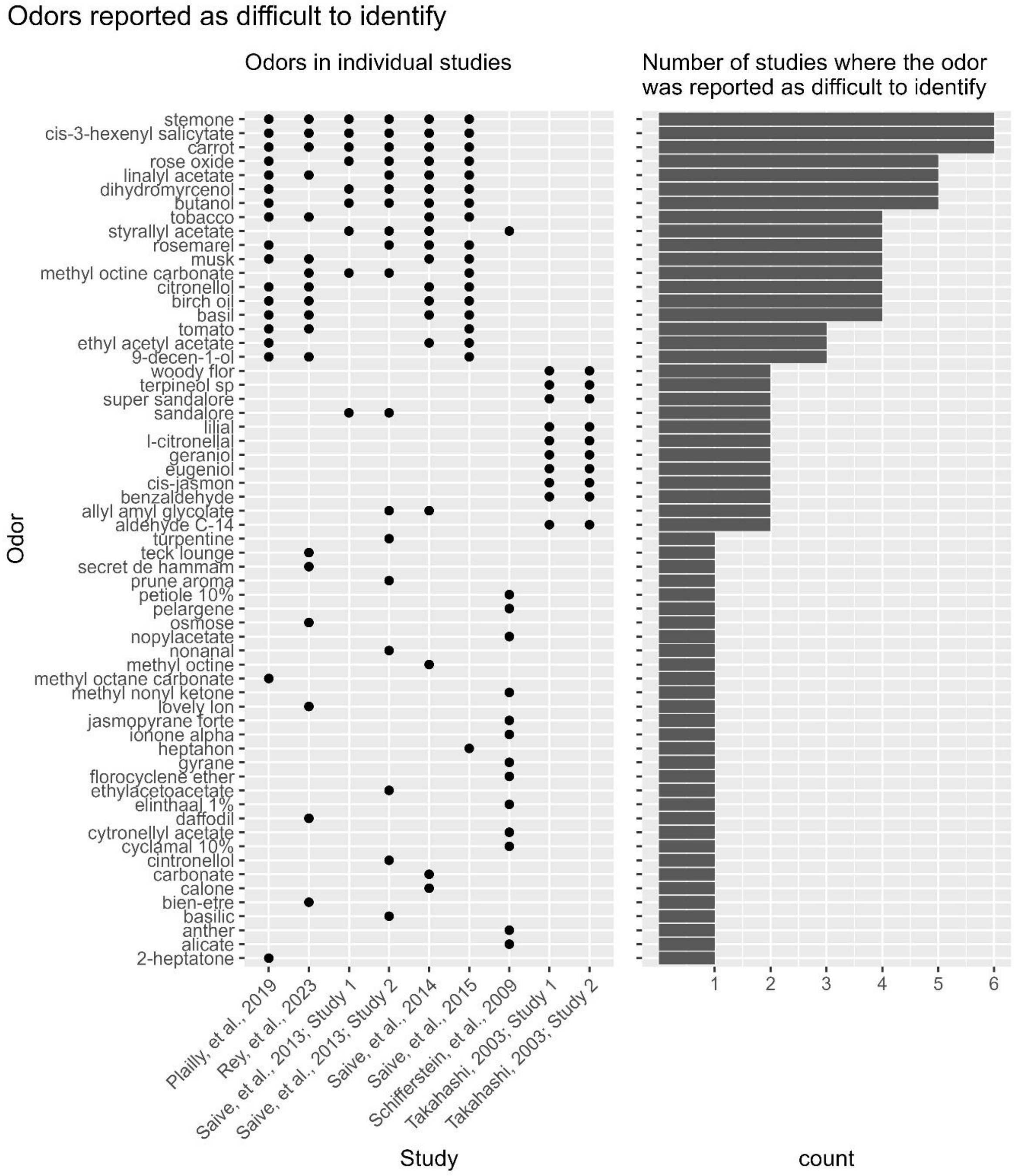

Supplement figure 6. List of the odors reported as easy to identify (per study and count).

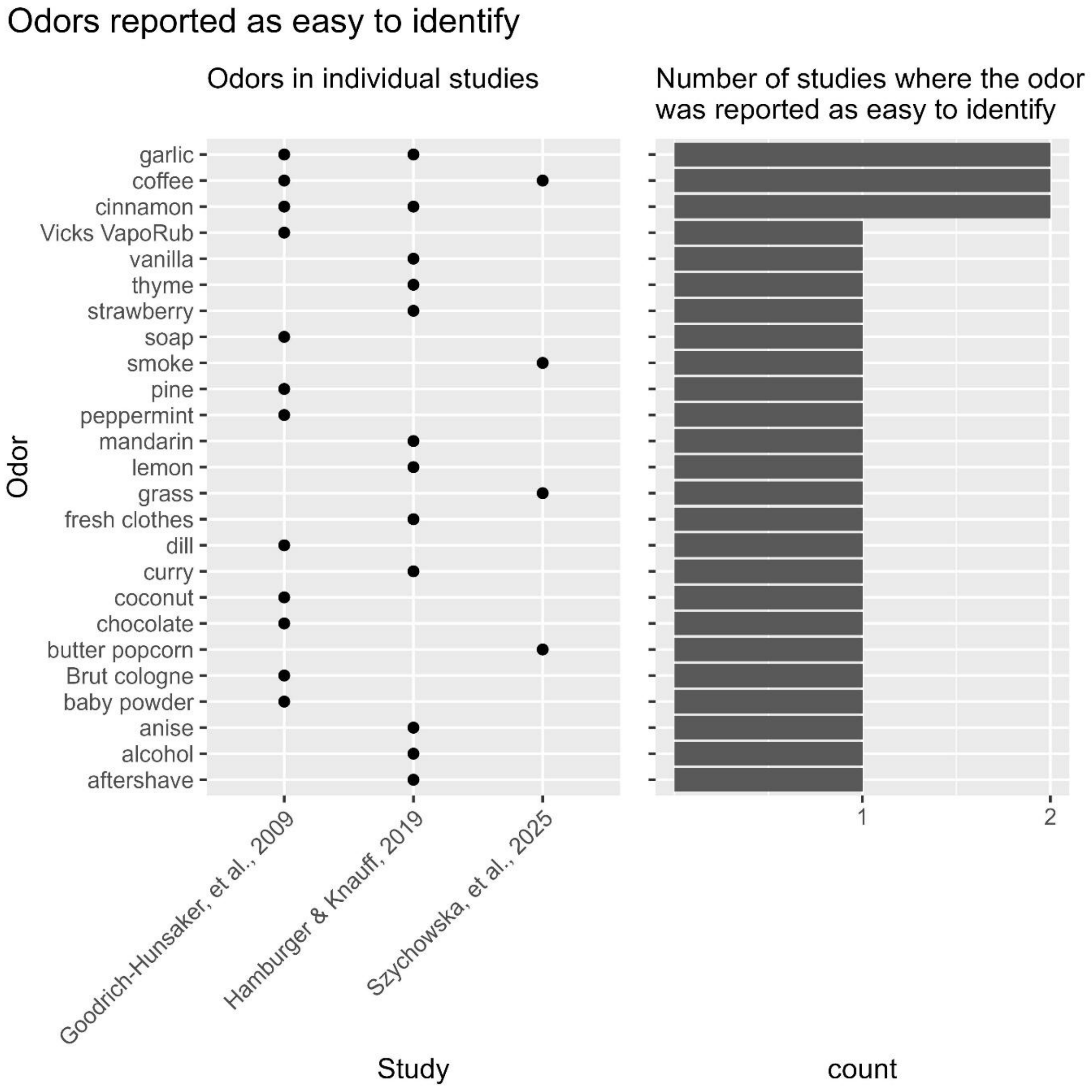

Supplement figure 7. List of the odors with identifiability not reported (per study and count).

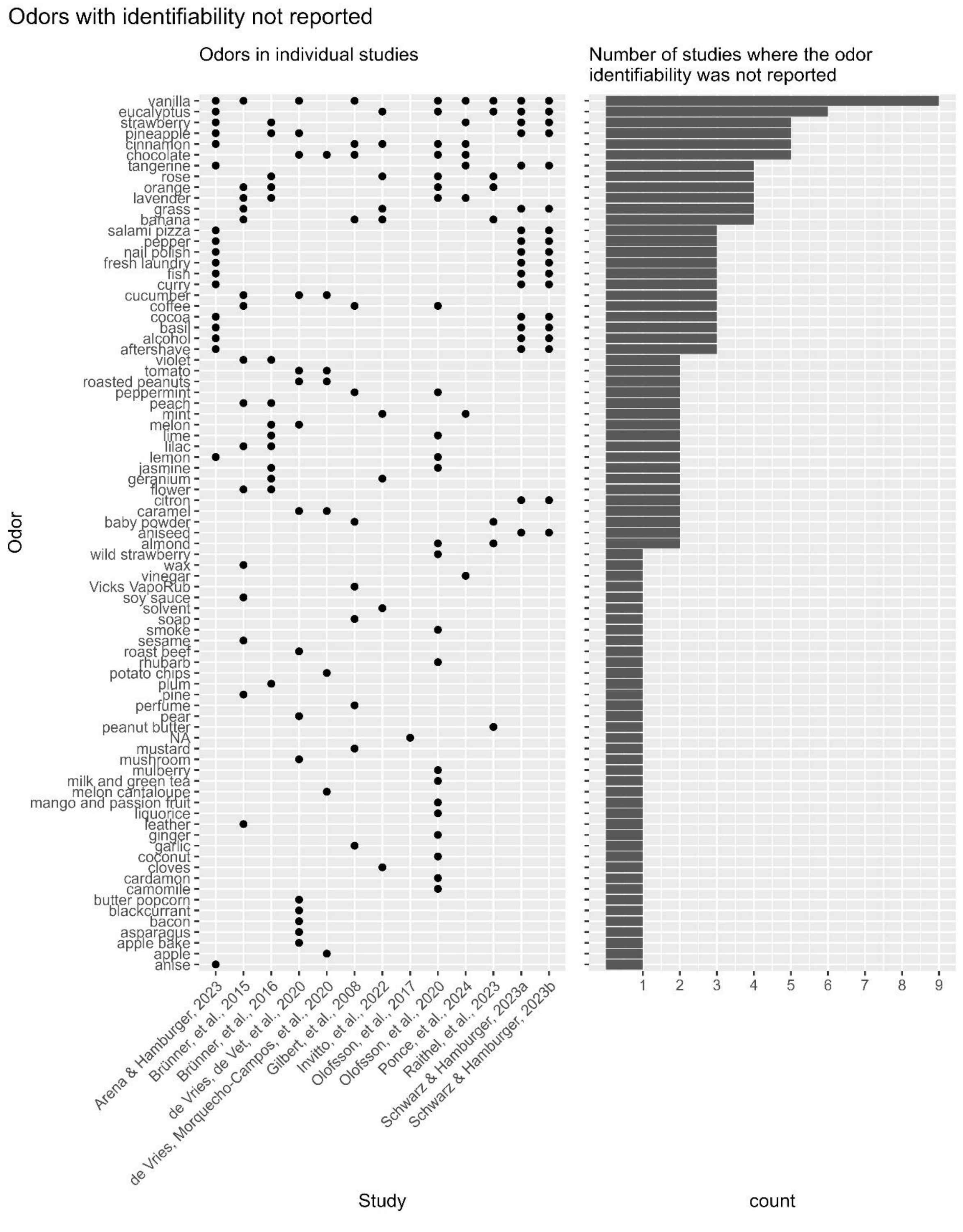

Supplement: Supplementary file 4 — Supplementary Material 4 [file 41598_2025_25503_MOESM4_ESM.pdf]
